# Untargeted metabolomics approach and molecular networking analysis reveal changes in chemical composition under the influence of altitudinal variation in bamboo species

**DOI:** 10.3389/fmolb.2023.1192088

**Published:** 2023-05-24

**Authors:** Luis Carlos Chitiva, Hair Santiago Lozano-Puentes, Ximena Londoño, Tiago F. Leão, Mónica P. Cala, Eduardo Ruiz-Sanchez, Lucía Ana Díaz-Ariza, Juliet A. Prieto-Rodríguez, Ian Castro-Gamboa, Geison M. Costa

**Affiliations:** ^1^ Department of Chemistry, Faculty of Sciences, Pontificia Universidad Javeriana, Bogotá, Colombia; ^2^ Institute of Chemistry, São Paulo State University (UNESP), Araraquara, Brazil; ^3^ Department of Biology, Faculty of Sciences, Pontificia Universidad Javeriana, Bogotá, Colombia; ^4^ Faculty of Agricultural Sciences, Universidad Nacional de Colombia, Palmira, Colombia; ^5^ Metabolomics Core Facility-MetCore, Universidad de los Andes, Bogotá, Colombia; ^6^ Department of Botany and Zoology, Universidad de Guadalajara, Jalisco, México

**Keywords:** bamboo, *Guadua*, altitudinal variation, flavonoids, cinnamic acid derivatives, untargeted metabolomics, GNPS, natural products

## Abstract

Bamboo species have traditionally been used as building material and potential source of bioactive substances, as they produce a wide variety of phenolic compounds, including flavonoids and cinnamic acid derivatives that are considered biologically active. However, the effects of growth conditions such as location, altitude, climate, and soil on the metabolome of these species still need to be fully understood. This study aimed to evaluate variations in chemical composition induced by altitudinal gradient (0–3000 m) by utilizing an untargeted metabolomics approach and mapping chemical space using molecular networking analysis. We analyzed 111 samples from 12 bamboo species collected from different altitudinal ranges using liquid chromatography coupled with quadrupole time-of-flight mass spectrometry (LC-QTOF-MS). We used multivariate and univariate statistical analyses to identify the metabolites that showed significant differences in the altitude environments. Additionally, we used the Global Natural Products Social Molecular Networking (GNPS) web platform to perform chemical mapping by comparing the metabolome among the studied species and the reference spectra from its database. The results showed 89 differential metabolites between the altitudinal ranges investigated, wherein high altitude environments significantly increased the profile of flavonoids. While, low altitude environments significantly boosted the profile of cinnamic acid derivatives, particularly caffeoylquinic acids (CQAs). MolNetEnhancer networks confirmed the same differential molecular families already found, revealing metabolic diversity. Overall, this study provides the first report of variations induced by altitude in the chemical profile of bamboo species. The findings may possess fascinating active biological properties, thus offering an alternative use for bamboo.

## 1 Introduction

Bamboo has gained immense value in recent times due to its versatile applications in construction, food, cosmetics, and medicine (Liese et al., 2015; Ming et al., 2017; Chongtham and Bisht, 2020). Bamboo is a rich source of active compounds such as flavonoids, phenolic acid derivatives, alkaloids, terpenes, and essential oils (Coffie et al., 2014; Gomez et al., 2021; Gagliano et al., 2022; Indira et al., 2022; Okido et al., 2022; Cheng et al., 2023) that are characterized by their antioxidant (Panche et al., 2016; Speisky et al., 2022), antimicrobial (Xie et al., 2015), antiviral (Badshah et al., 2021), and anti-inflammatory (Maleki et al., 2019) properties, among others. While most chemical and biological studies have focused on the Asian continent (Clark et al., 2015; Gagliano et al., 2022; Tamang et al., 2022), there is a pressing need to deepen research on Neotropical bamboos to identify alternative uses and create additional value for these species.

The metabolic profile of plants can be affected by several environmental factors, such as temperature, light, ultraviolet radiation levels, precipitation, humidity, nutrients, and altitude (Khalil et al., 2020; Kumari et al., 2022). Additionally, genetic factors, including the presence of genes that control metabolite biosynthesis and the participation of enzymes in different biosynthetic pathways, have been shown to contribute to this variation (Dhami and Mishra, 2015; Sampaio et al., 2016; Pant et al., 2021). However, few studies have investigated changes in the chemical composition of bamboo species due to environmental or genetic effects.

Recent studies have revealed that certain changes can significantly impact the biological potential of bamboo. Specifically, research has shown that seasonal and altitudinal variation in *Sasa argenteastriatus* (*Pleioblastus argenteostriatus* (Regel) Nakai) and *S. quelpaertensis* Nakai leaves is positively correlated with an increase in the content of phenolic and flavonoid compounds, with chlorogenic acid, isoorientin, and vitexin being the most notable compounds exhibiting significant changes (Ni et al., 2012; Ko et al., 2018). Another study on *Indocalamus latifolius* (Keng) McClure evaluated the impact of altitude on the chemical composition of flavonoids, phenols, and triterpenes, demonstrating that an increase in altitude led to the accumulation of metabolites and a subsequent increase in antioxidant potential (Ni et al., 2013). Based on these findings, we anticipate observing a similar correlation between the increase in phenolic compound content and the species in our study, providing valuable insights for improving crop production and obtaining biologically active metabolites.

Metabolomics is a valuable tool for evaluating metabolic changes in various biological matrices caused by environmental or genetic factors. To analyze large amounts of metabolites in a biological sample, different analytical platforms are currently used. When combined with multivariate analysis, these platforms can identify differentially expressed metabolites, helping to elucidate possible metabolic pathways affected (Verpoorte et al., 2010; Shen et al., 2023). To integrate other platforms and complement the global analysis of the metabolome in bamboo species, we used Global Natural Products Social Molecular Networking (GNPS), a novel platform that facilitates the creation of molecular networks to analyze mass spectrometry (MS/MS) data sets, providing a comprehensive visualization of the chemical space (Wang et al., 2016; Aron et al., 2020; Ramabulana et al., 2021). To our knowledge, this is the first report that applies the untargeted metabolomics approach and molecular networking analysis to the study of the chemical composition of bamboo species under the influence of altitude. This study aimed to evaluate the variations in chemical composition under the influence of an altitudinal gradient (0–3000 m) by utilizing an untargeted metabolomics approach and the mapping of the chemical diversity using molecular networking analysis of the global metabolome in bamboo species.

## 2 Materials and methods

### 2.1 Study design

**FIGURE 1:**
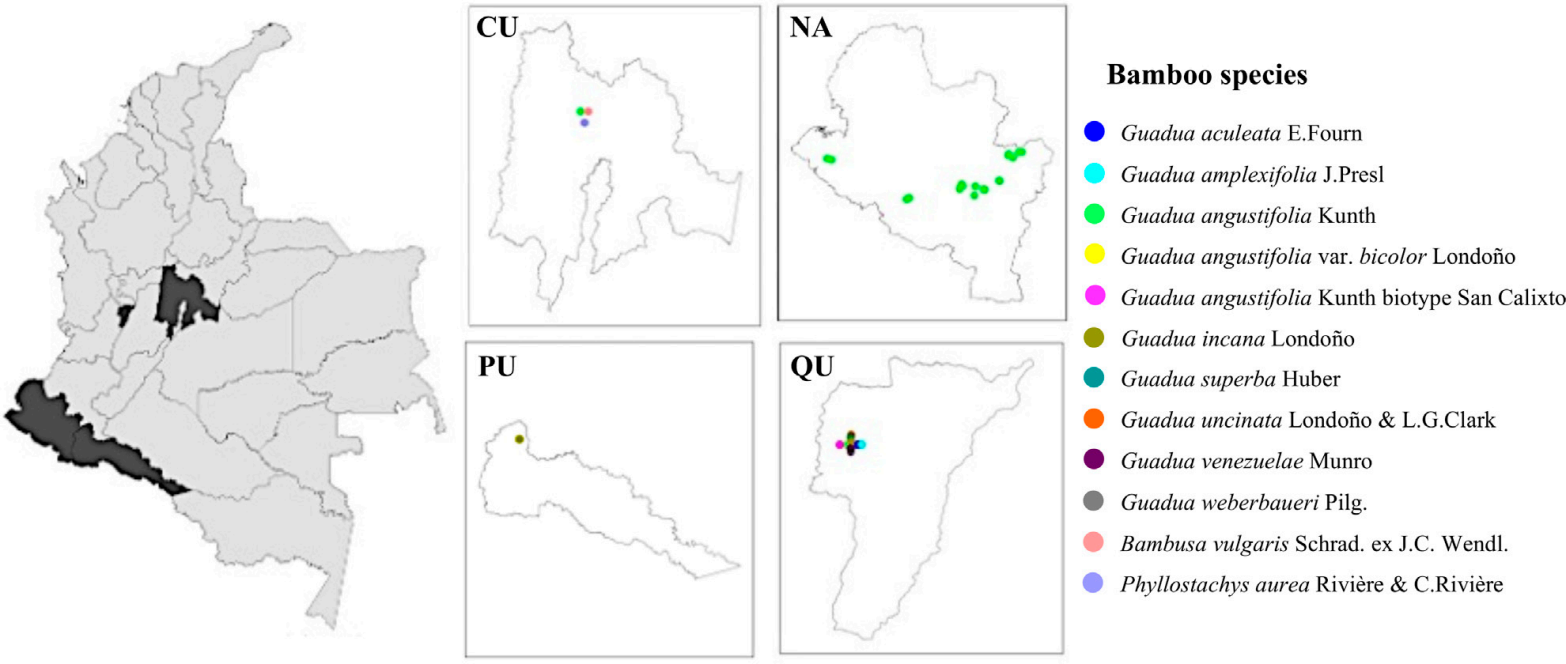
Geographical distribution map showing the bamboo species studied. Each mark corresponds to the place of collection of each specimen.

A completely random sampling method was implemented for the metabolomic study by selecting different collection sites in Colombian locations [Cundinamarca (CU), Nariño (NA), Putumayo (PU), and Quindío (QU)] situated at different altitudes, ranging from (0–3000 m). The geographical distributions of the collected species are shown in Figure 1. All samples were collected from natural bamboo in the period 2020 to 2022 (more details are provided in Supplementary Table S1).

### 2.2 Plant material collection and identification

We collected a total of 111 leaf samples from the upper branches of 12 bamboo species, which were distributed across 40 different altitudes and collected under the same conditions. Harvested bamboo leaves were immediately air-dried at room temperature and then ground into powder for extraction. Based on altitude, we divided the 111 leaf samples into two groups: the low altitude group (∼0–1500 m; *n* = 40) and high altitude group (∼1500–3000 m; *n* = 71), as indicated in Table 1. At least three individuals (*n* = 3) were collected at each collection site, and voucher specimens were deposited in the (HPUJ) Herbarium of the Pontificia Universidad Javeriana.

**TABLE 1:** Classification of bamboo species according to altitudinal range.

Plant scientific name	Sampling location	Altitude (m)	Group
*G. aculeata* E.Fourn.	Quindío, Montenegro	1256	Low
*G. amplexifolia* J.Presl	Quindío, Montenegro	1256	Low
*G. angustifolia* Kunth	Nariño, Tumaco	18	Low
	Nariño, Tumaco	21	Low
	Nariño, Ricaurte	1053	Low
	Nariño, Ricaurte	1089	Low
	Quindío, Montenegro	1256	Low
	Cundinamarca, Pacho	1343	Low
	Nariño, Samaniego	1478	Low
	Nariño, Samaniego	1565	High
	Nariño, Samaniego	1588	High
	Nariño, San Lorenzo	1595	High
	Nariño, La Unión	1598	High
	Nariño, Consacá	1606	High
	Nariño, Samaniego	1606	High
	Nariño, La Unión	1610	High
	Nariño, La Unión	1631	High
	Nariño, San Lorenzo	1713	High
	Nariño, Sandoná	1720	High
	Nariño, Sandoná	1744	High
	Nariño, San Lorenzo	1779	High
	Nariño, San Lorenzo	1808	High
	Nariño, San Lorenzo	1826	High
	Nariño, Chachagüí	1857	High
	Nariño, San Lorenzo	1876	High
	Nariño, San Lorenzo	1930	High
	Nariño, San Lorenzo	1970	High
	Nariño, La Florida	2089	High
	Nariño, La Florida	2122	High
	Nariño, La Florida	2137	High
*G. angustifolia* var. *bicolor* Londoño	Quindío, Montenegro	1256	Low
*G. angustifolia* Kunth biotype San Calixto	Quindío, Montenegro	1256	Low
*G. incana* Londoño	Putumayo, Mocoa	604	Low
	Quindío, Montenegro	1256	Low
*G. superba* Huber	Quindío, Montenegro	1256	Low
*G. uncinata* Londoño & L.G.Clark	Quindío, Montenegro	1256	Low
*G. venezuelae* Munro	Quindío, Montenegro	1256	Low
*G. weberbaueri* Pilg.	Quindío, Montenegro	1256	Low
*B. vulgaris* Schrad. ex J.C. Wendl.	Cundinamarca, Pacho	1343	Low
*P. aurea* Rivière & C.Rivière	Quindío, Montenegro	1256	Low

### 2.3 Metabolite extraction and sample preparation

To extract the plant material (leaves), 300 mg of dried and ground samples were mixed with 10 mL of a solvent containing chloroform, methanol, and water (in a ratio of 5:2.5:2.5 *v*/*v*/*v*). The resulting mixture was then vortexed for 1 min, sonicated for 20 min, and centrifuged at 5000 rpm and 20°C for 10 min. The liquid supernatant was filtered using PTFE syringe filters with a pore size of 0.22 µm (Thermo Scientific, Rockwood, TN), and stored at −80°C until used for metabolomics analysis.

### 2.4 Untargeted metabolomics using LC-QTOF-MS

Untargeted metabolomics was conducted using an Agilent Infinity 1260 HPLC system coupled to an Agilent 6545 quadrupole time-of-flight (QTOF) mass spectrometer equipped with electrospray ion source (Waldbronn, Germany). 5 μL of the extracts were injected on a C18 column (Kinetex 100 × 2.1 mm, 2.6 µm) at 30°C. The mobile phase employed in LC analyses was composed of 0.1% formic acid in Milli-Q water (*v/v*) (Solvent A) and acetonitrile (Solvent B) with a constant flow of 400 μL/min. The gradient was set as follows: 3% for 1 min; 3%–97% B in 15 min; 97% B for 2 min; the column was re-equilibrated for 6 min at the initial conditions. Full-scan MS1 and MS/MS spectra were acquired. Data mass spectra were acquired in negative ionization mode (ESI^−^), in a mass range of *m/z* 80–1700 Da in data-dependent acquisition (DDA) mode. The QTOF instrument was operated in the 4 GHz (high resolution) mode. The parameters used for data acquisition were set as follows: nitrogen used as nebulizer gas with pressure at 52 psi, a capillary voltage of 3000 V, ion source temperature of 250°C, dry gas flow at 12 L/min, and acquisition rate of one spectrum per second. MS/MS fragmentation was performed using a collision-induced dissociation energy of 20 eV. Throughout the analysis, two reference masses were used for mass correction: *m/z* 112.9856 [C_2_O_2_F_3_(NH_4_)] and *m/z* 1033.9881(C_18_H_18_O_6_N_3_P_3_F_24_).

### 2.5 Quality control samples

To evaluate system performance and reproducibility in sample analysis, multiple QC samples were created by pooling and mixing equal volumes of each extracted sample. To assess the instrument’s robustness, pooled QC samples were injected before the sample analysis until system equilibration was achieved and after every ten randomized sample injections.

### 2.6 Metabolomics data processing

The LC-QTOF-MS raw data sets were processed using Agilent MassHunter Workstation Profinder software (B.10.0, Agilent Technologies) to extract molecular features for deconvolution, alignment, and integration. The data were manually inspected to eliminate noise and unrelated ions, and a presence filter was applied. For statistical analysis, features that were present in 100% of the samples for each altitudinal group and had a coefficient of variation (CV) of less than 20% in the QC were selected.

### 2.7 Statistical analysis

Univariate and multivariate analyses were conducted using MatLab (R2019b, MathWorks, Inc., Natick) and SIMCA 14.0 software (Umetrics, Umeå, Sweden), respectively. The multivariate analysis generated PCA and OPLS-DA models, which were validated using cross-validation less than 0.05 and evaluated based on R^2^X (change in X explained by the model), R^2^Y (the total of Y explained), and Q^2^ (sum parameter in cross-validation). The significantly differential metabolites were identified by calculating the variable importance in the projection (VIP) greater than 1, with jackknife confident interval (JK) not including zero combined with FC > 2.0 or FC < 0.5. The annotated metabolites and their peak area were organized in a table (.csv), which was uploaded to MetaboAnalyst 5.0 software for statistical, functional, and integrative analysis of metabolomics data (https://www.metaboanalyst.ca/). The software was used for visualization using heatmap clustering (Pang et al., 2022), normalized by Pareto scaling. Additionally, univariate analysis was performed to determine the *p*-value features.

### 2.8 Metabolite identification

Differential metabolite annotation was conducted by considering the precision of the mass (maximum error of mass 10 ppm), isotopic pattern distribution, and adduct formation, using different public online databases such as METLIN (https://metlin.scripps.edu/), KEGG (https://genome.jp/kegg), HMDB (https://hmdb.ca/), PubChem (https://pubchem.ncbi.nlm.nih.gov/) and ChEBI (https://www.ebi.ac.uk/chebi/) through the CEU Mass Mediator (https://ceumass.eps.uspceu.es/) tool. The identity of the metabolites was confirmed through MS/MS analysis, which included the use of MS-DIAL 4.80 (https://prime.psc.riken.jp/compms/msdial/main.html), MS-FINDER 3.52 (https://prime.psc.riken.jp/compms/msfinder/main.html), CFM-ID 4.0 (https://cfmid.wishartlab.com/) for *in silico* mass spectral fragmentation, GNPS web platform (https://gnps.ucsd.edu/ProteoSAFe/static/gnps-splash.jsp) and manual interpretation with the Agilent Mass Hunter Qualitative Analysis software (version 10.0). The metabolites were identified according to the metabolomics standards initiative (Schymanski et al., 2014).

### 2.9 Global natural products social molecular networking (GNPS) web platform workflow description

**FIGURE 2:**
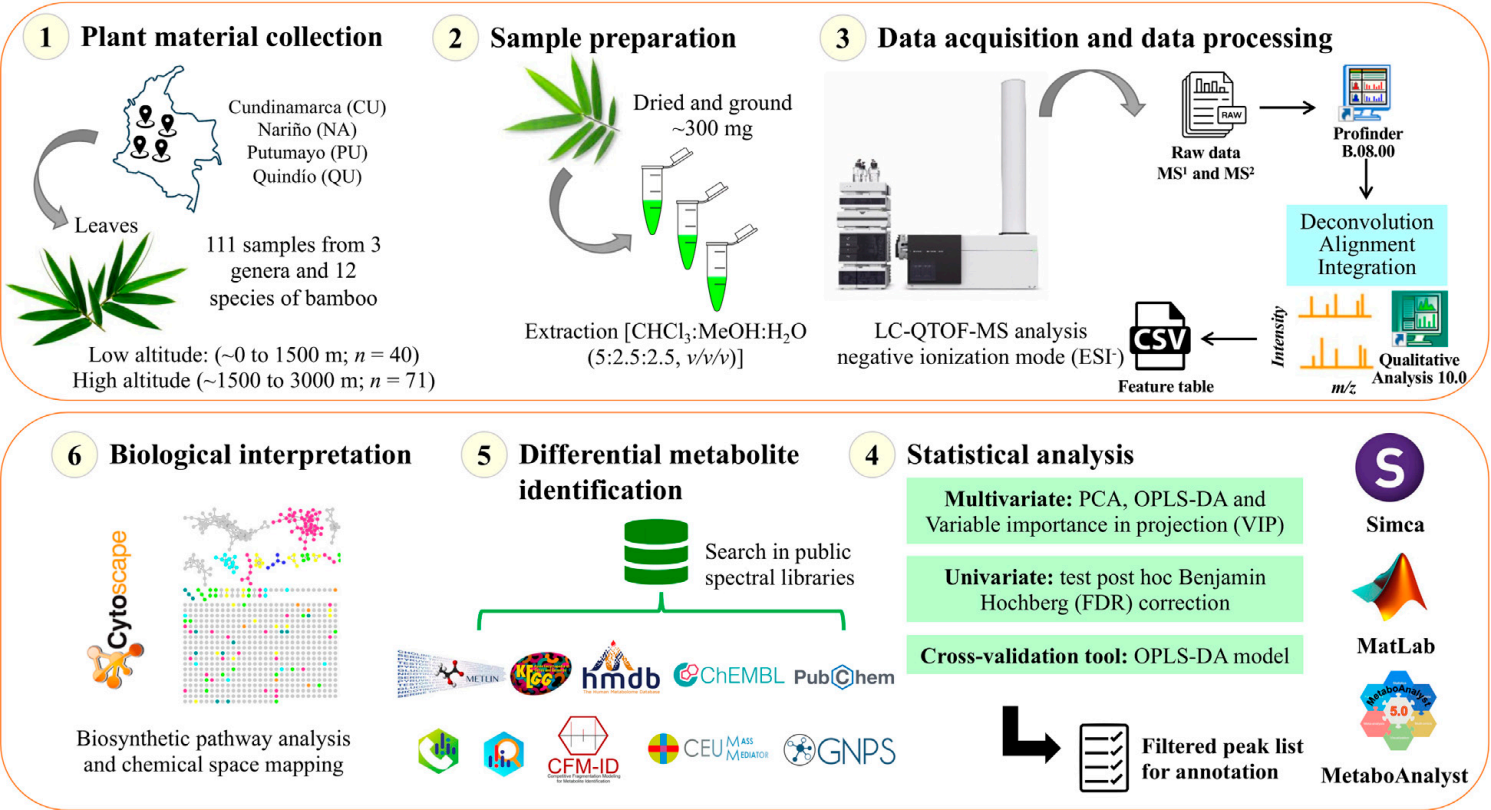
Schematic of the experiment analytical workflow used in this study.

A molecular network was created using the online workflow (https://ccms-ucsd.github.io/GNPSDocumentation/) on the GNPS website (https://gnps.ucsd.edu/). The precursor ion mass tolerance was set to 0.02 Da and an MS/MS fragment ion tolerance of 0.02 Da. A network was then created where edges were filtered to have a cosine score above 0.6 and more than four matched peaks. Further, edges between two nodes were kept in the network if and only if each of the nodes appeared in each other’s respective top 10 most similar nodes. Finally, the maximum size of a molecular family was set to 0, and the lowest-scoring edges were removed from molecular families until the molecular family size was below this threshold. The spectra in the network were then searched against GNPS spectral libraries. All matches kept between network spectra and library spectra were required to have a score above 0.6 and at least four matched peaks (Wang et al., 2016). To enhance chemical structural information within the molecular network, information from *in silico* structure annotations from GNPS Library Search, Network Annotation Propagation, Dereplicator were incorporated into the network using the GNPS MolNetEnhancer workflow (https://ccms-ucsd.github.io/GNPSDocumentation/molnetenhancer/). Chemical class annotations were performed using the ClassyFire chemical ontology (Djoumbou Feunang et al., 2016; Mohimani et al., 2017; Da Silva et al., 2018; Ernst et al., 2019). The attribute table of the generated nodes was visualized in the Cytoscape software to analyze the molecular network. The data used for the analysis of molecular networks were deposited in the MassIVE Public GNPS database (http://massive.ucsd.edu) with the accession number MSV000090298. The workflow used in this study is summarized in Figure 2.

## 3 Results

### 3.1 Chemical variation of bamboo species presented between low and high altitudes

**FIGURE 3:**
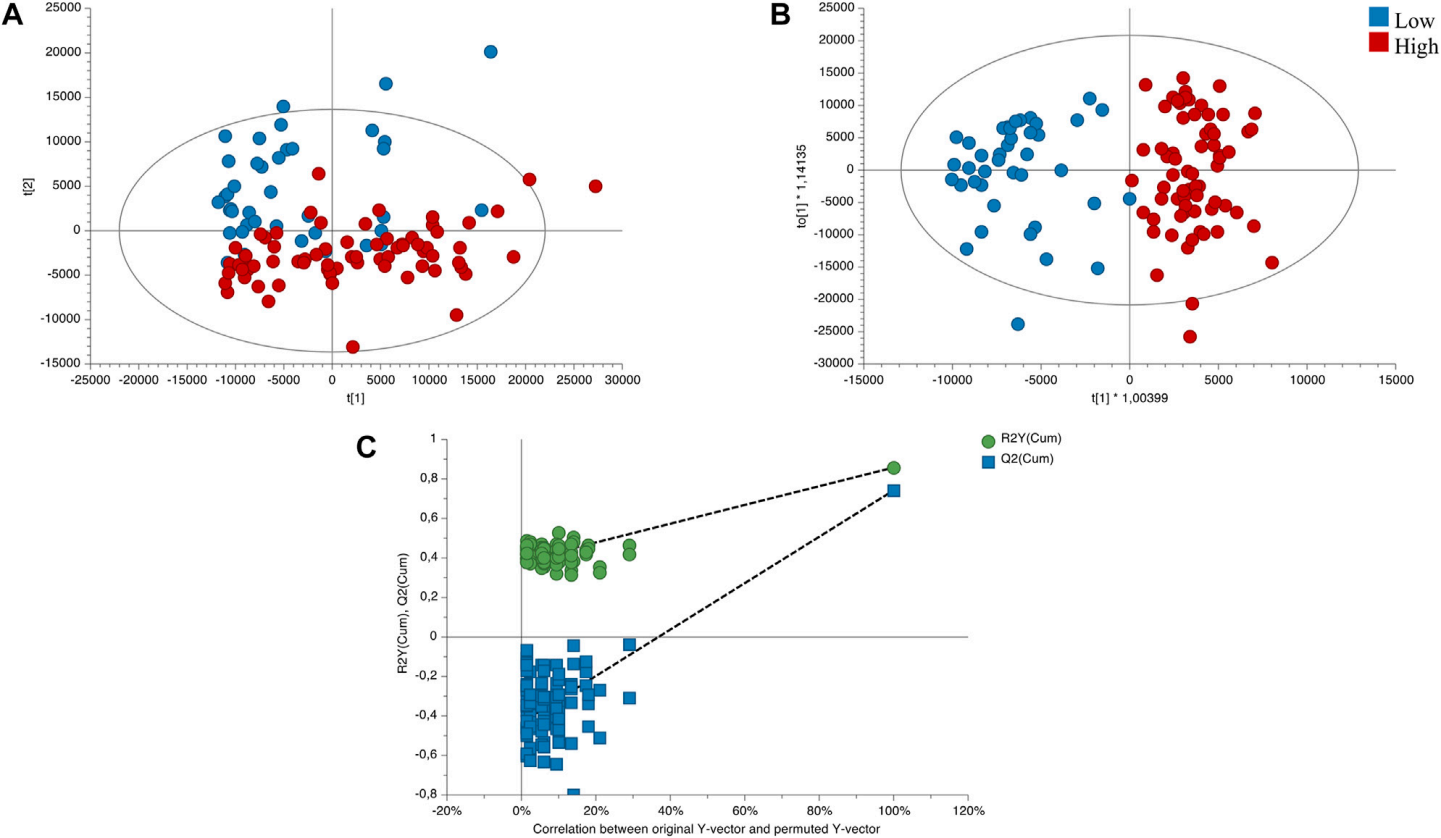
Score plots of the influence of direction compared low/high altitudes. **(A)** PCA score plot of total samples (R^2^X_(cum)_: 0.813; Q^2^_(cum)_: 0.54). **(B)** OPLS-DA score plot of the low vs. high altitudes sample (R^2^X_(cum)_: 0.494; R^2^Y_(cum)_:0.858; Q^2^_(cum)_:0.742; CV Anova: 9.61549e-27). **(C)** Cross-validation plot of the OPLS-DA model with 200 permutation test. Low altitude (∼0–1500 m; *n* = 40) and high altitude (∼1500–3000 m; *n* = 71).

The quality and stability of the instrument were assessed by conducting a principal component analysis (PCA) on the quality control samples. Supplementary Figure S1 shows a clustering of the QC samples, which indicates the analytical platform’s reliability and the data’s validity. To account for altitudinal variation, we performed PCA and orthogonal partial least squares discriminant analysis (OPLS-DA) models on samples classified into two altitude ranges. Based on this classification, we developed an unsupervised PCA model to observe trends between the two altitude groups. As depicted in Figure 3A, the PCA model showed an initial exploration of the data set, evidencing certain trends between the variables of the altitude groups. Additionally, we constructed a supervised OPLS-DA model to differentiate between low and high altitude groups, with appropriate quality parameters, demonstrating complete separation of the groups. (Figure 3B). This allowed us to identify the significant variables that contributed to the separation between the groups, yielding a total of 89 significant variables with values (false discovery rate, FDR) < 0.05, VIP >1, and FC > 2.0 (or <0.5), as identified according to the metabolomics standards initiative (Schymanski et al., 2014). To validate the OPLS-DA model, we conducted permutation analysis, plotting *R* ^2^ and Q^2^ of 200 permutation tests, as shown in Figure 3C.

Table 2 presents the differential metabolites (89) that belong to various families, such as flavonoids (48%), fatty acids (19%), cinnamic acid derivatives (11%), unknowns (6%), peptides (3%), glycosylated lignans (2%), alkaloids (2%), carboxylic acids (1%), phenols (1%), carbohydrates (1%), steroids (1%), glycosylated stilbenes (1%) and prenolipids (1%). The largest group of differentially expressed metabolites comprises flavonoids which are the most frequently reported metabolites for these species. Many metabolites in this group showed an upward trend at higher altitudes, potentially contributing to the medicinal potential of these species.

**TABLE 2:** Significantly differential metabolites found the altitudinal variation.

Compound name	Molecular formula	Molecular weight (g/mol)	RT (min)	Mass error (ppm)	Adduct	ID level	^a^CV for QC (%)	Low vs. high altitude samples		
								^b^FC	^c^VIP	^d^ *p-*value with FDR
Flavonoids										
Luteolin 6-*C*-glucoside 8-*C*-arabinoside	C_27_H_30_O_16_	610.1534	5.17	1	[M-H]^−^	2	0.64	0.67	3.26	2.41E-02
Quercetin 3,7-dirhamnoside	C_27_H_30_O_15_	594.1585	5.39	1	[M-H]^−^	2	1.73	0.65	3.71	2.81E-04
Kaempferol-3-*O*-rutinoside	C_27_H_30_O_15_	594.1585	6.09	4	[M-H]^−^	4	0.86	0.77	2.34	1.70E-02
Vitexin 6''-(3-hydroxy-3-methylglutarate)	C_27_H_28_O_14_	576.1479	6.86	1	[M-H-H_2_O]^−^	3	3.42	0.45	3.03	6.10E-05
Maysin	C_27_H_28_O_14_	576.1479	5.86	1	[M-H]^−^	2	3.53	0.53	3.58	1.47E-04
Cassiaoccidentalin B	C_27_H_28_O_14_	576.1479	6.72	2	[M-H]^−^	2	1.76	0.58	2.70	2.91E-04
Nicotiflorin	C_27_H_30_O_15_	594.1585	5.96	0	[M-H]^−^	2	2.95	0.68	1.88	8.91E-03
Scutellarein 4′-methyl ether 7-glucuronide	C_22_H_20_O_12_	476.0955	6.44	1	[M-H]^−^	2	1.46	0.44	2.68	3.25E-06
Denticulaflavonol	C_35_H_42_O_6_	558.2981	12.92	9	[M+Cl]^−^	2	0.94	0.26	2.03	7.02E-12
Apigenin 7-[6''-(3-Hydroxy-3-methylglutaryl)glucoside]	C_27_H_28_O_14_	576.1479	8.29	5	[M-H-H_2_O]^−^	4	1.00	0.61	2.21	1.23E-03
Quercetin 3-(2″,3″,4″-triacetylgalactoside)	C_27_H_26_O_15_	590.1272	6.90	1	[M-H-H_2_O]^−^	3	0.74	0.35	2.33	5.17E-07
Hosloppin	C_22_H_16_O_7_	392.0896	8.82	7	[M+HCOO-H]^−^	2	0.62	0.68	1.54	1.25E-03
Vitexin 2″-*O*-rhamnoside*	C_27_H_30_O_14_	578.1636	5.96	3	[M-H]^−^	2	0.58	0.67	1.87	1.41E-03
Allivicin	C_27_H_30_O_16_	610.1534	5.81	1	[M-H]^−^	2	1.38	0.75	2.35	2.99E-02
Paniculatin	C_27_H_30_O_15_	594.1585	6.86	0	[M-H-H_2_O]^−^	4	2.88	0.50	2.42	1.24E-03
6″-*O*-(3-Hydroxy-3-methylglutaroyl)astragalin	C_27_H_28_O_15_	592.1428	6.21	1	[M-H]^−^	2	1.60	0.58	1.31	3.73E-03
Baicalin	C_21_H_18_O_11_	446.0849	6.18	3	[M-H]^−^	4	2.31	0.47	1.59	2.05E-03
4′-*O*-Methylneobavaisoflavone 7-*O*-(2″-*p*-coumaroylglucoside)	C_36_H_36_O_11_	644.2258	6.14	8	[M-H]^−^	3	0.63	2.14	1.37	8.96E-03
Apigenin 7-[rhamnosyl-(1->2)-galacturonide]	C_27_H_28_O_15_	592.1428	6.21	2	[M-H]^−^	4	2.60	0.66	1.09	2.01E-03
Isorhamnetin 3-galactoside-7-rhamnoside	C_28_H_32_O_16_	624.1690	5.52	2	[M-H]^−^	2	0.71	0.67	1.47	8.10E-03
Violanthin	C_27_H_30_O_14_	578.1636	6.51	4	[M-H]^−^	4	1.05	0.56	1.27	2.10E-05
Eruberin B	C_30_H_40_O_15_	640.2367	6.23	2	[M-H]^−^	3	1.81	5.15	1.07	9.72E-04
Kaempferol 7-sophoroside	C_27_H_30_O_16_	610.1534	5.17	6	[M-H]^−^	2	0.26	0.74	2.44	4.45E-02
Vicenin 2*	C_27_H_30_O_15_	594.1585	5.96	1	[M-H]^−^	2	3.76	0.74	1.74	2.13E-02
Thonningianin B	C_35_H_30_O_17_	722.1483	9.07	3	[M+HCOO-H]^−^	2	0.86	0.58	1.49	1.25E-04
Bracteoside	C_22_H_20_O_12_	476.0955	6.01	2	[M-H]^−^	4	5.58	0.48	1.07	4.91E-05
Orientin 2″-rhamnoside	C_27_H_30_O_15_	594.1585	6.09	1	[M-H]^−^	2	0.77	0.74	2.47	6.33E-03
7,8,3′,4′-Tetrahydroxyflavanone 7-(2,4,6-triacetylglucoside)	C_27_H_28_O_14_	576.1479	6.72	1	[M-H]^−^	2	1.66	0.57	2.72	2.80E-04
Epigallocatechin 3-gallate	C_22_H_18_O_11_	458.0849	7.93	2	[M-H]^−^	2	2.83	0.45	1.15	3.29E-06
Glychalcone A	C_22_H_22_O_5_	366.1467	1.34	4	[M-H]^−^	4	1.85	0.48	1.29	6.72E-08
Quercetin 3-(3″,6″-diacetylgalactoside)	C_25_H_24_O_14_	548.1166	6.94	6	[M-H-H_2_O]^−^	3	1.39	0.52	1.06	9.13E-05
2″,4″-Diacetylafzelin	C_25_H_24_O_12_	516.1268	6.79	1	[M-H]^−^	3	0.39	0.73	2.54	3.65E-03
Epigallocatechin 3-*O*-caffeate	C_24_H_20_O_10_	468.1057	8.30	5	[M+HCOO-H]^−^	3	1.06	0.48	1.16	1.05E-06
Cyanidin 3-rutinoside	C_27_H_31_O_15_	595.1663	6.72	8	[M-H-H_2_O]^−^	4	1.72	0.73	1.29	1.24E-02
5,7,3′,4′-Tetrahydroxyflavanone 7-alpha-L-arabinofuranosyl-(1->6)-glucoside	C_26_H_30_O_15_	582.1585	5.17	2	[M+HCOO-H]^−^	4	0.24	0.63	2.60	2.76E-03
Isoorientin 2″-*O*-rhamnoside	C_27_H_30_O_15_	594.1585	5.86	2	[M-H]^−^	4	1.15	0.60	2.82	1.20E-03
Neosaponarin	C_27_H_30_O_15_	594.1585	6.72	2	[M-H]^−^	4	0.99	0.64	2.92	1.85E-03
Chamaemeloside	C_27_H_28_O_14_	576.1479	6.08	1	[M-H]^−^	2	1.39	0.59	1.68	3.16E-05
5′,5‴,8,8″-Tetrahydroxy-3′,3‴,4′,4‴,7′,7″-hexamethoxy-5,5″-biflavan	C_36_H_38_O_12_	662.2363	5.90	8	[M-H-H_2_O]^−^	3	0.33	0.61	1.45	1.39E-05
Kaempferol 7-neohesperidoside	C_27_H_30_O_15_	594.1585	6.09	1	[M-H]^−^	2	0.85	0.72	2.56	4.44E-03
Saponarin*	C_27_H_30_O_15_	594.1585	5.40	2	[M-H]^−^	2	1.32	0.69	2.78	7.22E-04
Astragalin 7-rhamnoside	C_27_H_30_O_15_	594.1585	6.54	1	[M-H-H_2_O]^−^	3	1.66	0.51	1.61	5.64E-05
Apigenin 7-glucuronide-4′-rhamnoside	C_27_H_28_O_15_	592.1428	6.54	1	[M-H-H_2_O]^−^	3	1.95	0.50	1.64	5.20E-05
Fatty acids										
TriHODE	C_18_H_32_O_5_	328.2250	8.07	1	[M-H]^−^	4	0.36	0.65	3.90	2.34E-08
Sativic acid	C_18_H_36_O_6_	348.2512	8.45	1	[M-H-H_2_O]^−^	4	0.26	0.61	3.96	4.91E-13
Coriolic acid	C_18_H_32_O_3_	296.2351	11.70	1	[M-H]^−^	2	0.75	0.35	2.40	7.02E-12
HoTrE	C_18_H_30_O_3_	294.2195	11.20	1	[M-H]^−^	2	0.76	0.33	2.44	1.84E-12
Dodecanedioic acid	C_12_H_22_O_4_	230.1518	6.43	0	[M-H]^−^	2	0.45	0.67	1.88	1.95E-04
TriHOME	C_18_H_34_O_5_	330.2406	8.88	2	[M-H]^−^	4	1.37	0.48	1.30	3.66E-07
Lauric acid	C_12_H_22_O_3_	214.1569	8.39	1	[M-H]^−^	2	0.64	0.43	1.14	2.13E-07
Undecylenic acid	C_11_H_20_O_2_	184.1463	6.70	1	[M+HCOO-H]^−^	4	0.61	0.68	1.26	1.89E-03
HpODE	C_18_H_32_O_4_	312.2301	10.50	1	[M-H]^-^	4	1.25	0.46	1.02	1.74E-07
HpOTrE	C_18_H_30_O_4_	310.2144	10.09	1	[M-H]^−^	4	1.74	0.41	1.03	2.56E-07
Undecenoic acid	C_11_H_20_O_2_	184.1463	8.07	1	[M+HCOO-H]^−^	4	0.87	0.65	1.23	3.88E-07
Cascarillic acid	C_11_H_20_O_2_	184.1463	8.45	1	[M+HCOO-H]^−^	4	0.88	0.53	1.43	5.49E-12
Sorbic acid	C_6_H_8_O_2_	112.0524	3.33	1	[M-H-H_2_O]^−^	3	1.11	0.61	1.24	9.88E-07
Malyngic acid	C_18_H_32_O_5_	328.2250	9.08	2	[M-H]^−^	4	9.26	0.60	1.36	9.87E-07
Fulgidic acid	C_18_H_32_O_5_	328.2250	8.55	1	[M-H]^−^	4	1.53	0.56	1.01	1.06E-05
Hydroxyjasmonic acid	C_12_H_18_O_4_	226.1205	7.50	1	[M-H]^−^	2	0.83	0.61	1.04	3.25E-06
Norlinolenic acid	C_17_H_28_O_2_	264.2089	9.08	2	[M+HCOO-H]^−^	4	0.89	0.41	1.72	7.02E-12
Cinnamic acid derivatives										
*O*-Caffeoylquinic acid	C_25_H_24_O_12_	516.1268	6.79	5	[M-H]^−^	2	0.26	0.77	2.16	5.80E-03
*p*-Coumaroylquinic acid	C_16_H_18_O_8_	338.1002	4.97	2	[M-H]^−^	4	1.22	4.38	1.15	2.61E-06
*O*-Feruloyl-beta-D-glucose	C_16_H_20_O_9_	356.1107	4.24	1	[M-H-H_2_O]^−^	4	1.39	2.39	1.14	2.12E-03
Dihydrocaffeic acid 3-*O*-glucuronide	C_15_H_18_O_10_	358.0900	5.12	7	[M+HCOO-H]^−^	2	0.60	0.73	1.37	1.12E-02
*O*-Feruloylgalactarate	C_16_H_18_O_11_	386.0849	5.83	7	[M+HCOO-H]^−^	2	0.45	0.62	2.09	1.80E-06
Caffeic acid 3-glucoside	C_15_H_18_O_9_	342.0951	7.31	8	[M+HCOO-H]^−^	4	1.05	0.66	1.49	3.44E-03
Dihydroferulic acid 4-*O*-glucuronide	C_16_H_20_O_10_	372.1056	5.30	1	[M-H]^−^	4	0.83	2.96	1.51	3.60E-07
1-Caffeoyl-4-deoxyquinic acid	C_16_H_18_O_8_	338.1002	5.28	1	[M-H]^−^	4	9.71	2.79	1.04	1.32E-02
Quinic acid	C_7_H_12_O_6_	192.0634	4.73	2	[M-H]^−^	2	0.91	5.05	1.42	3.14E-03
1-*O*-Sinapoylglucose	C_17_H_22_O_10_	386.1213	5.48	1	[M-H]^−^	4	0.88	6.16	1.24	8.80E-09
Unknowns										
Unknown 1 (396.036@4.25)	—	—	4.25	—	—	5	1.89	0.32	2.27	1.54E-07
Unknown 2 (586.0623@8.9)	—	—	8.90	—	—	5	1.77	0.67	1.49	4.69E-04
Unknown 3 (572.0832@8.44)	—	—	8.44	—	—	5	0.45	0.79	1.02	1.65E-02
Unknown 4 (255.989@8.9)	—	—	8.90	—	—	5	1.36	0.66	1.11	8.14E-05
Unknown 5	C_27_H_36_O_12_	—	6.14	—	—	4	0.88	2.10	1.36	1.02E-02
Unknown 6	C_27_H_36_O_12_	—	5.90	—	—	4	0.39	0.61	1.80	2.56E-05
Peptides										
Tripeptide 1	C_19_H_25_N_3_O_7_	407.1692	4.65	6	[M-H]^−^	3	1.16	9.13	1.62	2.22E-09
Tripeptide 2	C_19_H_25_N_3_O_7_	407.1692	4.55	7	[M-H]^−^	3	1.46	10.31	1.61	1.56E-10
Tripeptide 3	C_15_H_20_N_4_O_6_	352.1383	4.81	5	[M-H]^−^	4	1.09	4.36	1.89	1.84E-12
Glycosylated lignans										
Prupaside	C_27_H_36_O_12_	552.2207	5.90	1	[M+HCOO-H]^−^	3	0.40	0.61	1.88	2.37E-05
Citrusin B	C_27_H_36_O_13_	568.2156	6.27	2	[M+HCOO-H]^−^	4	1.00	0.58	1.02	6.10E-05
Alkaloids										
2′-Norberbamunine	C_35_H_38_N_2_O_6_	582.2730	8.51	5	[M+HCOO-H]^−^	4	1.30	0.39	1.07	4.43E-12
Vomilenine	C_21_H_22_N_2_O_3_	350.1630	4.88	8	[M+Cl]^−^	4	0.50	1.36	1.44	1.04E-02
Carboxylic acids										
3,4,5-trihydroxy-6-(2-hydroxy-6-methoxyphenoxy)oxane-2-carboxylic acid	C_13_H_16_O_9_	316.0794	2.06	2	[M-H]^−^	4	0.88	2.14	1.54	5.09E-04
Phenols										
Phenol glucuronide	C_12_H_14_O_7_	270.0740	2.06	0	[M+HCOO-H]^−^	4	0.90	2.14	1.54	5.10E-04
Carbohydrates										
Ribulose	C_5_H_10_O_5_	150.0528	0.60	0	[M-H]^−^	4	0.76	0.75	1.14	1.21E-03
Steroids										
Physalin L	C_28_H_32_O_10_	528.1995	1.54	3	[M-H]^−^	2	1.11	0.40	1.08	1.07E-07
Glycosylated stilbenes										
Piceatannol 4′-galloylglucoside	C_27_H_26_O_13_	558.1373	7.69	2	[M-H]^−^	3	2.60	0.36	1.23	2.24E-05
Prenolipids										
Auxin b	C_18_H_30_O_4_	310.2144	9.08	1	[M-H]^−^	4	1.13	0.41	1.81	7.07E-12

^a^ CV, coefficient of variation in the metabolites in the QC samples.

^b^ FC, fold change in the comparison (average in low altitude/average in high altitude).

^c^ VIP, variable importance in projection.

^d^ *p*-value corresponding to the *p* values calculated by the Benjamini–Hochberg false discovery rate *post hoc* correction (FDR <0.05); *Metabolites annotated with GNPS. RT: retention time; Confidence levels in annotation were as following: Level 1: Confirmed structure, Level 2: Probable structure, Level 3: Tentative candidate(s), Level 4: Unequivocal molecular formula, Level 5: Exact mass (Schymanski et al., 2014).

### 3.2 Molecular networks and heatmap analyses reveal the chemical variation under the influence of altitudinal variation

**FIGURE 4:**
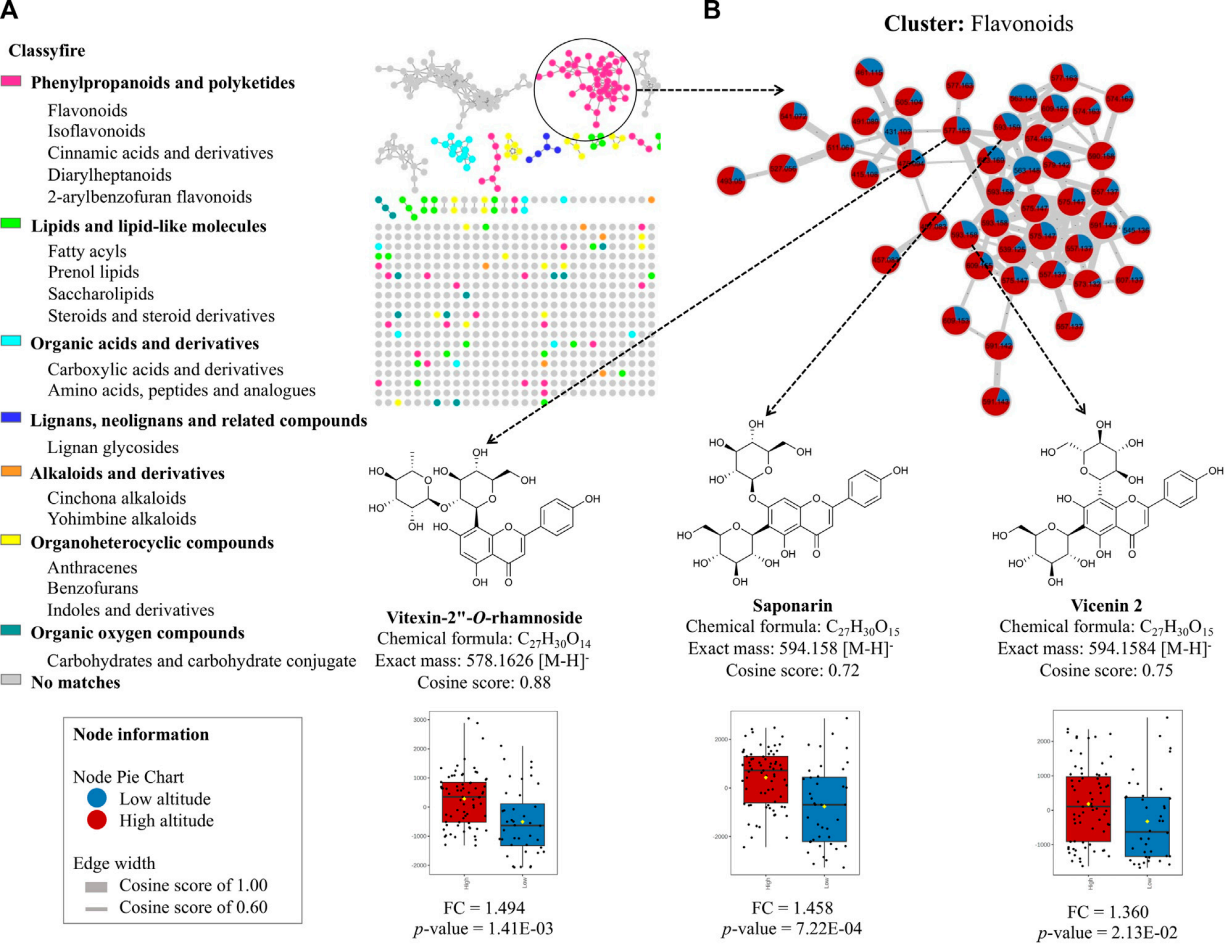
Molecular network analysis obtained from GNPS platform online. **(A)** Identification of major classes of chemical constituents of bamboo species using MolNetEnhancer technique and GNPS molecular networking. Color of the node is set according to the chemical class using “Classyfire”. **(B)** Molecular network of the flavonoid cluster comparing low altitude (blue) and high altitude (red).

To explore the leading chemical classes, we created a molecular network using the GNPS platform, which enabled us to visualize the chemical space of the metabolome in these species. Figure 4A illustrates the molecular network analyzed in the MolNetEnhancer platform, revealing the classes of MF present in the metabolome. We identified seven groups of molecular families, including phenylpropanoids and polyketides, lipids and lipid-like molecules, organic acid derivatives, lignans, neolignans and related compounds, alkaloids, and derivatives, organoheterocyclic compounds, and oxygenated organic compounds. However, due to the complexity of the metabolome in these species and the limitations of the spectral libraries, many of the nodes did not match any spectral reference. Therefore, it is crucial to continue exploring the chemical composition of these species, which is still limited in the literature. Notably, when we created a molecular network of the flavonoid cluster and compared the low vs. high altitude groups of the annotated compounds, we found that vitexin 2″-*O*-rhamnoside (FC = 1.494; *p*-value = 1.41E-03), saponarin (FC = 1.458; *p*-value = 7.22E-04), and vicenin 2 (FC = 1.360; *p*-value = 2.13E-02) (Supplementary Figures S2A–C) exhibited positive correlations at higher altitudes, confirming the statistical analysis performed previously (Figure 4B).

**FIGURE 5:**
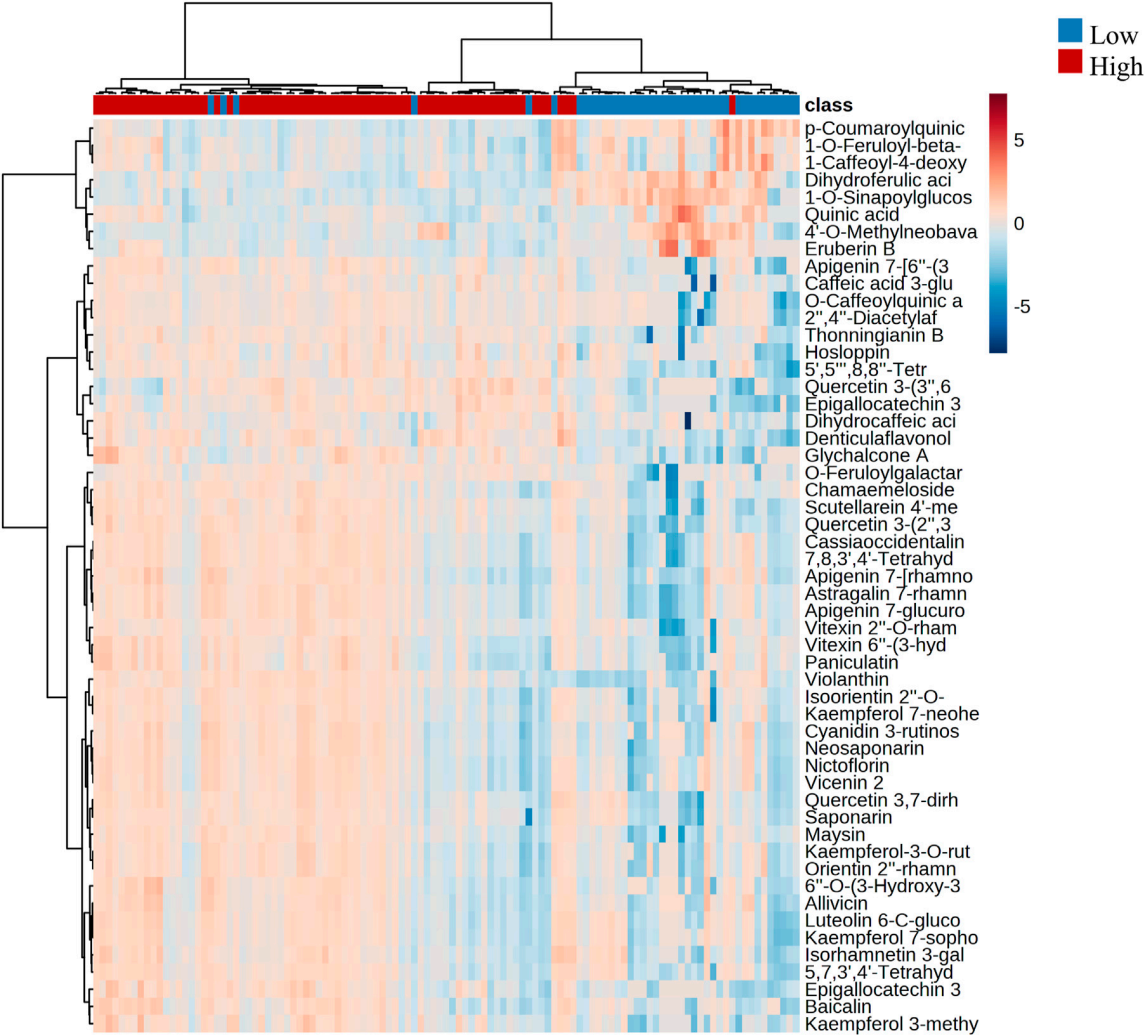
Hierarchical clustering with heatmap illustrating the differences in the metabolite abundance between low and high altitudes. The *x*-axis shows the clustering of all the samples, and the *y*-axis shows the clustering of the annotated flavonoids and cinnamic acid derivatives.

By conducting hierarchical clustering analysis and generating a heatmap based on the two most relevant groups of annotated metabolites that exhibited significant changes with altitude (flavonoids and cinnamic acid derivatives), we identified two distinct groups that were associated with altitude. We found that each group exhibited different patterns in terms of chemical composition and levels of presence and abundance. Figure 5 depicts the clustering of the two altitudinal ranges evaluated, with the first cluster corresponding to the high altitude samples that exhibited a high accumulation of flavonoids, while the low altitude samples exhibited a high accumulation of cinnamic acid derivatives.

### 3.3 Changes in the concentration and analysis of biosynthesis pathways of the most relevant metabolites in bamboo species

**FIGURE 6:**
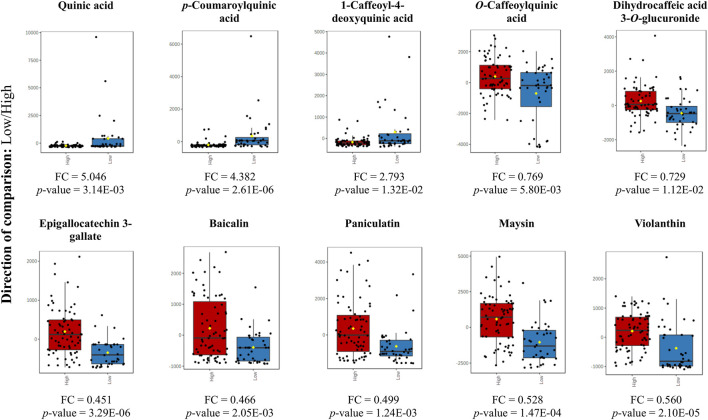
Box plot for the altered metabolites corresponding to cinnamic acid derivatives and flavonoids (*p*-value <0.05) at low altitude (blue) and high altitude (red). *Y*-axes are represented as relative units. The data were normalized with respect to the total spectral area. Bar charts show normalized values (mean ± one standard deviation). Boxes range from the 5% and 95% percentiles are indicated as error bars; individual data points are indicated by circles. The medians are indicated by horizontal lines within each box.

The box plot shows the differential metabolites for the two altitude groups. In Figure 6 we present the fold change of the main metabolites that exhibited a significant change. We found that cinnamic acid derivatives and flavonoids showed a significant change with the low and high altitude groups, respectively. Our study also highlighted the significance of quinic acid (QA) is a major differential metabolite, which acts as a crucial precursor in the biosynthetic pathway of phenylpropanoids and is essential in the production of a diverse array of phenolic compounds.

### 3.4 Molecular networking for chemical space mapping of the metabolome of bamboo species by MolNetEnhancer

**FIGURE 7:**
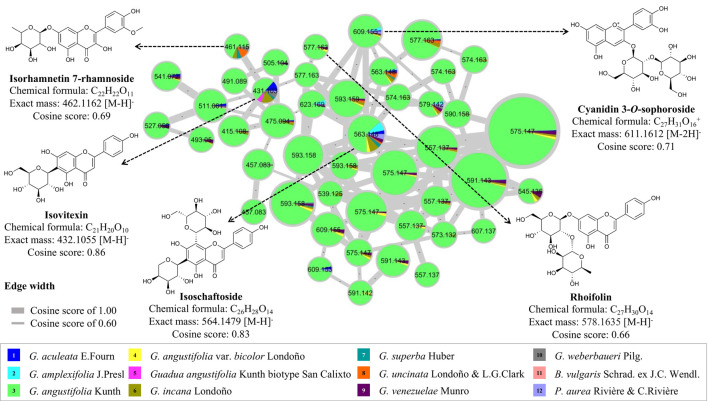
Molecular networking and dereplication of flavonoids comparing twelve bamboo species.

To complement the study on the chemical composition of the global metabolome of bamboo species, a molecular network was constructed using the GNPS platform to compare other types of flavonoids that were not statistically significant among the twelve species studied. Four flavonoid *C*-glycosides (isorhamnetin 7-rhamnoside, isovitexin, isoschaftoside, and rhoifolin) and one flavonoid *O*-glycoside (cyanidin 3-*O*-sophoroside) were annotated (Supplementary Figures S2D–H). It was observed that the *Guadua angustifolia* species was found to have an abundant profile of flavonoid *C*-glucosides, specifically of the compound isoschaftoside, which is functionally related to apigenin (Figure 7). In terms of flavonoid variation between species, isovitexin and isorhamnetin 7-rhamnoside were found in most species.

## 4 Discussion

**FIGURE 8:**
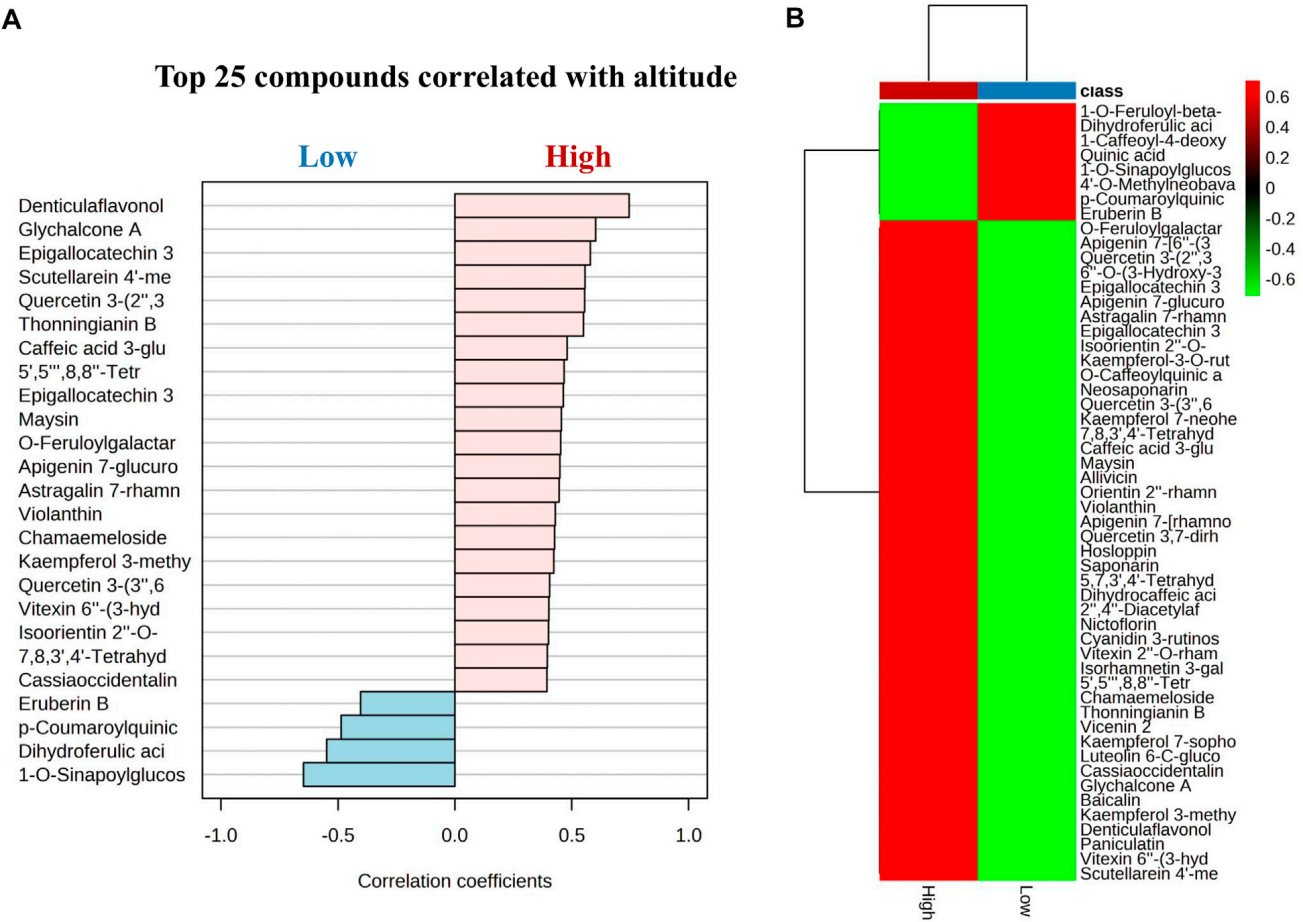
**(A)** Pearson’s correlations using pattern search of the top 25 metabolites between low and high altitude. **(B)** Cluster heatmap based on correlation between flavonoids and acid cinnamic derivatives and the altitude variable. The analysis shows a positive (red) and negative (green) correlation with a *p*-value = 0.05 for all the metabolites.

Our findings indicate that flavonoids significantly increased at high altitudes, while cinnamic acid derivatives exhibited an increasing trend at low altitudes. Notably, flavonoids from *G. angustifolia* showed a positive correlation with the high altitude group, as did cinnamic acid derivatives from *G. aculeata*, *G. amplexifolia*, *G. angustifolia* var. *bicolor*, *G. angustifolia* biotype San Calixto, *G. incana*, *G. superba*, *G. uncinata*, *G. venezuelae*, *B. vulgaris*, and *P. aurea* with low altitude (Figure 8). However, further research is needed to determine the exact nature of this correlation. The effect of altitudinal variation on the chemical composition of the leaves of bamboo species is poorly documented. Previous studies have suggested that plants grown at high altitudes tend to have higher levels of flavonoids compared to those grown at low altitudes (Wang et al., 2020; Zhou et al., 2021). This phenomenon can be attributed to an increase of in ultraviolet radiation, illumination time and the delay of the phenophase of the plant, along with elevation, which is a protective mechanism that plants use against unfavorable environmental conditions (Ni et al., 2013). In a recent study, Zhou et al. (2021) investigated the influence of an altitudinal gradient on the variation of flavonoids in *Agriophyllum squarrosum* and found that these metabolites were enriched at high altitudes. The study also demonstrated a strong positive correlation between the contents of flavonoids, such as quercetin, tricine, and rutin, and environmental variables, such as latitude, longitude, and precipitation gradients. These findings corroborate our results and provide valuable information on the variation in the chemical profile of bamboo under various abiotic factors such as altitude, temperature, light, and soil, etc.

The metabolic differences expressed in bamboo species between high and low altitudes provide valuable information about the ideal growing conditions to promote the production of phenolic compounds with important biological properties in medicine. Moreover, the bamboo leaves have added value as are agro-industrial residues generated in the construction industry. Our results suggest that the accumulation of flavonoids at high altitudes is due to the adaptability of bamboo species to environmental conditions. These giant grasses biosynthesize flavonoid-type phenolic compounds that are used as defence and potential antioxidants (Falcone Ferreyra et al., 2012; Panche et al., 2016; Wang et al., 2018). Notably, the phenylpropanoid biosynthesis pathway, which is the starting point for producing many essential compounds such as flavonoids, coumarins, lignans, and hydroxycinnamic acid conjugates (Fraser and Chapple, 2011), is possibly the pathway that has been altered considering the results of this study. Flavonoid production occurs mainly through a diverse biosynthetic pathway involving the shikimate pathway and polyketide pathways (Mouradov and Spangenberg, 2014; Liu et al., 2021). Interestingly, the phenylpropanoid biosynthetic pathway showed a significant change in the presence of QA, with a fold change of 5.05, VIP of 1.42, and a *p*-value with FDR 3.14E-03. QA is a metabolite that is closely related to the biosynthesis pathway of caffeoylquinic acids (CQAs) and are specialized bioactive metabolites that are derived from the phenylpropanoid biosynthesis pathway. Consequently, QA is a critical intermediate in the biosynthesis of many flavonoids through cinnamic acid, which is a necessary precursor (Alcázar Magaña et al., 2021; Liu et al., 2021). Furthermore, the study revealed interesting chemical diversity in these species, with a predominance of groups of metabolites that are flavonoids and cinnamic acid derivatives. The mapping of the chemical space (including information about known reference spectra) allowed for the visualization of a large part of the global chemical composition of these species. However, the GNPS platform had a low annotation rate, resulting in many no-matches.

To complement the study of changes in chemical composition under the influence of altitude, the molecular network was used to analyze the specific cluster for flavonoids, comparing low and high altitudes. The study showed that the flavonoids such as vitexin 2″-*O*-rhamnoside, saponarin, and vicenin 2 had a positive tendency to increase at high altitudes. This finding suggests a close relationship between the concentration of phenolic compounds (flavonoids and cinnamic acid derivatives) in bamboo species. The heatmap shows a clear difference in the content of flavonoids and cinnamic acid derivatives between the groups compared under the effect of the altitudinal gradient, which could serve as marker compounds for chemical classification. The metabolites exposed to the variable altitude showed a significant difference, indicating that environmental factors have previously influenced the genetic and chemical diversity of plants (Pacheco-Hernández et al., 2021).

The metabolome of various bamboo species was compared, it was found that *G. angustifolia* had a rich profile of *C*-glycoside flavonoids. Similar metabolites have been reported for other bamboo species such as *P. nigra* var. *henonis* (Zhang et al., 2008; Ibrahim et al., 2021) *P. pubescens* (Tanaka et al., 2014) and *B. vulgaris* (Akhtar and Patowary, 2022). Some flavonoids were found to be shared among other species, such as *G. aculeata*., *G. angustifolia*, *G. angustifolia* biotype San Calixto, *G. incana*, *G. uncinata,* and *G. venezuelae*. Out of the twelve species analyzed, *B. vulgaris* and *P. aurea* were found to be the most studied species at the chemical level in Asia. However, considering the limited information available in the literature and the results obtained from the metabolomic analysis, this study presents an opportunity to explore the metabolome of these species further, especially those belonging to the genus *Guadua* and distributed in the Neotropical region.

## 5 Conclusion

This study employed an untargeted metabolomics approach and molecular networking analysis to assess changes in the chemical composition of bamboo species due to variations in altitude. The study revealed that high altitude had a significant influence on the increase of flavonoid profiles, while low altitude led to an increase in cinnamic acid derivatives profiles. The molecular network analysis further demonstrated the diverse chemical composition of these species, including flavonoid glycosides, cinnamic acid derivatives, lignans, alkaloids, carbohydrates, and fatty acids. Conducting metabolomic studies on bamboo can provide a detailed understanding of its chemical composition and metabolic conservation, aiding in identifying patterns and trends in relation to environmental and cultivation factors. This information can help to enhance the production and quality of bamboo, identify bioactive compounds for natural health products, and improve sustainability, positively impacting the industry and the economy.

## Data Availability

The raw data supporting the conclusion of this article will be made available by the authors, without undue reservation.
