# Supplementary figures and images for: Untargeted metabolomics approach and molecular networking analysis reveal changes in chemical composition under the influence of altitudinal variation in bamboo species
Source: Front Mol Biosci. 2023 May 24;10:1192088. doi: 10.3389/fmolb.2023.1192088 (PMC10246775; doi:10.3389/fmolb.2023.1192088)

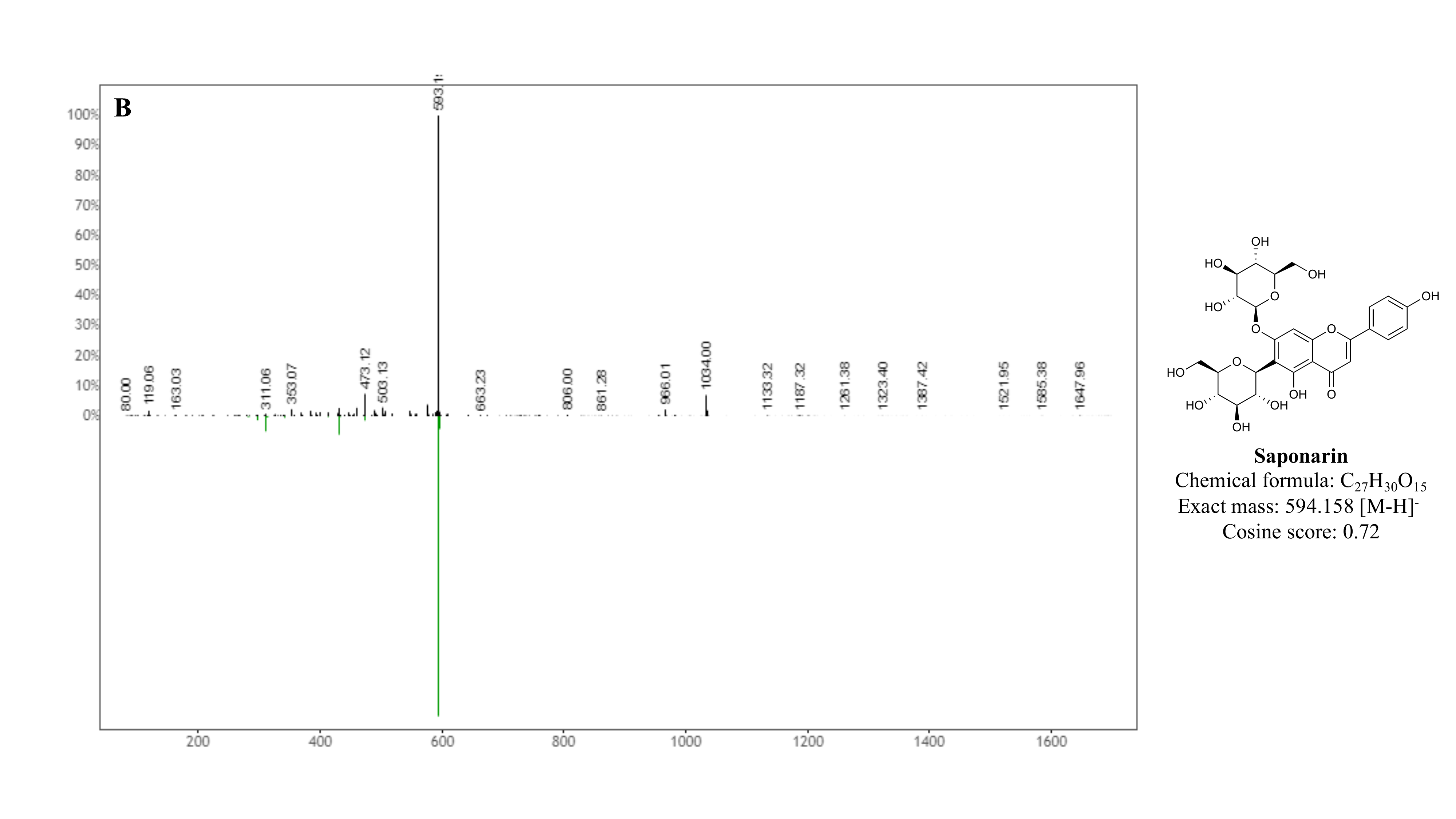

Supplement: Supplementary file 1 [file Image3.tiff]

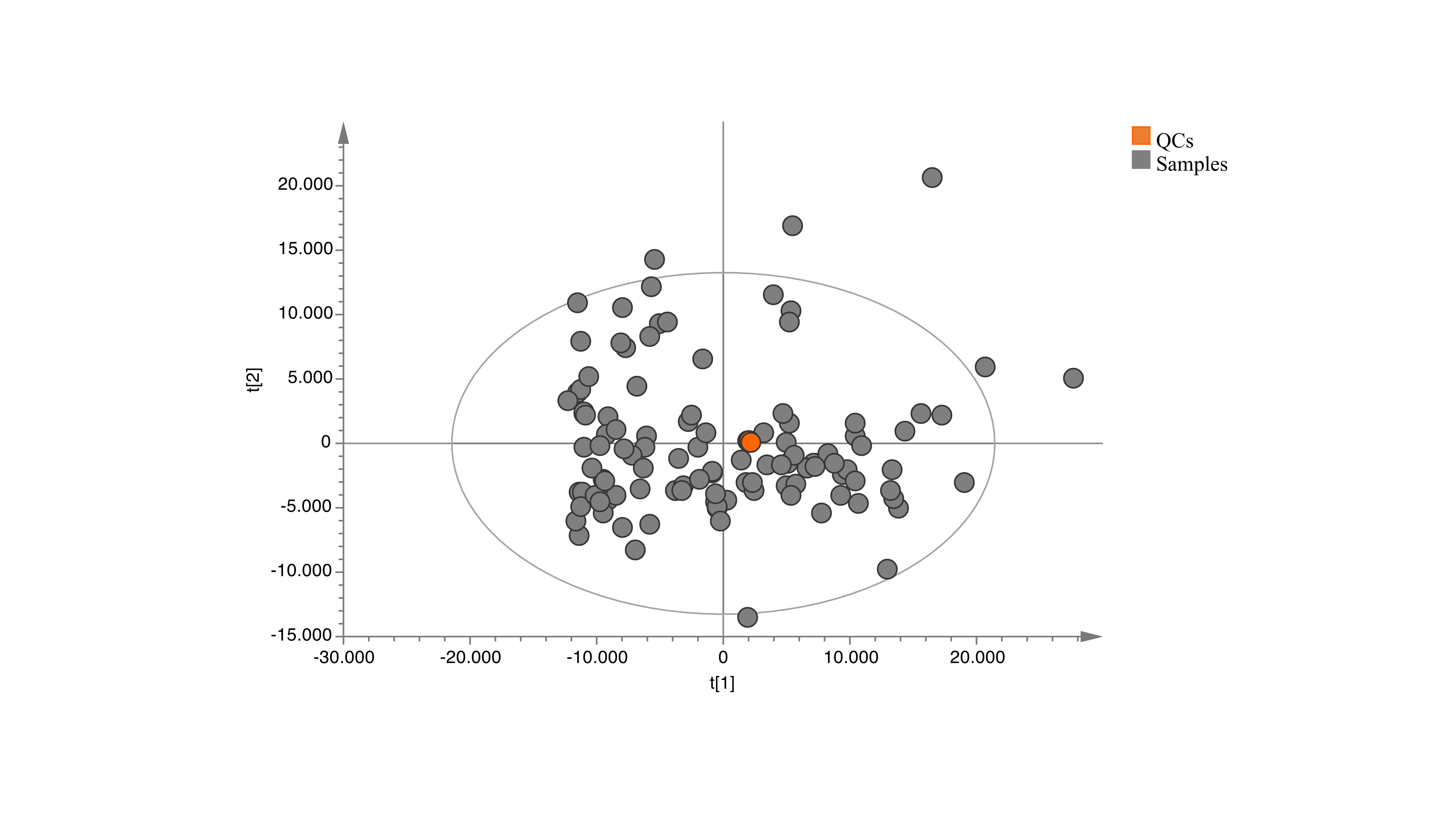

Supplement: Supplementary file 2 [file Image1.TIFF]

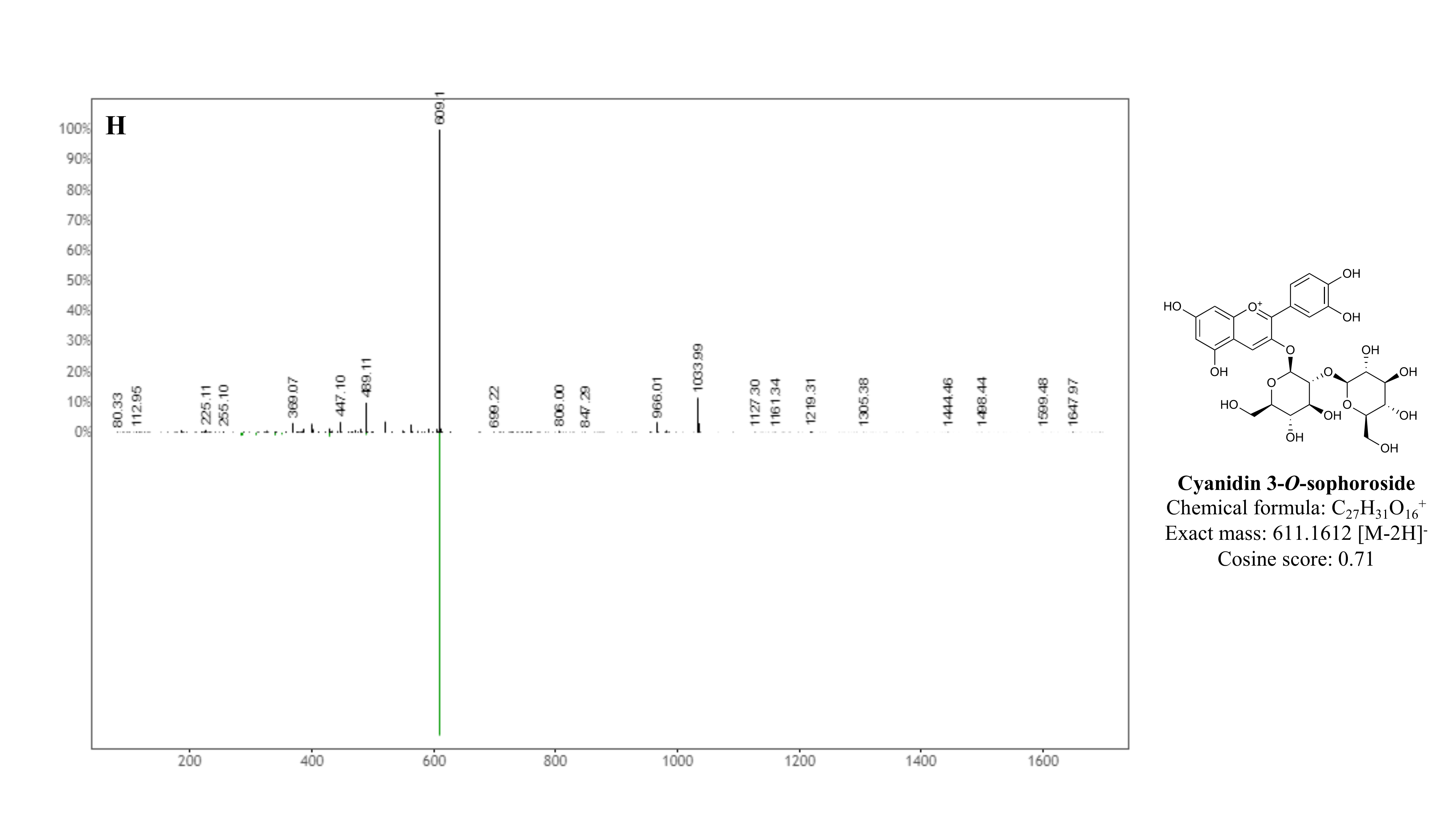

Supplement: Supplementary file 3 [file Image9.TIFF]

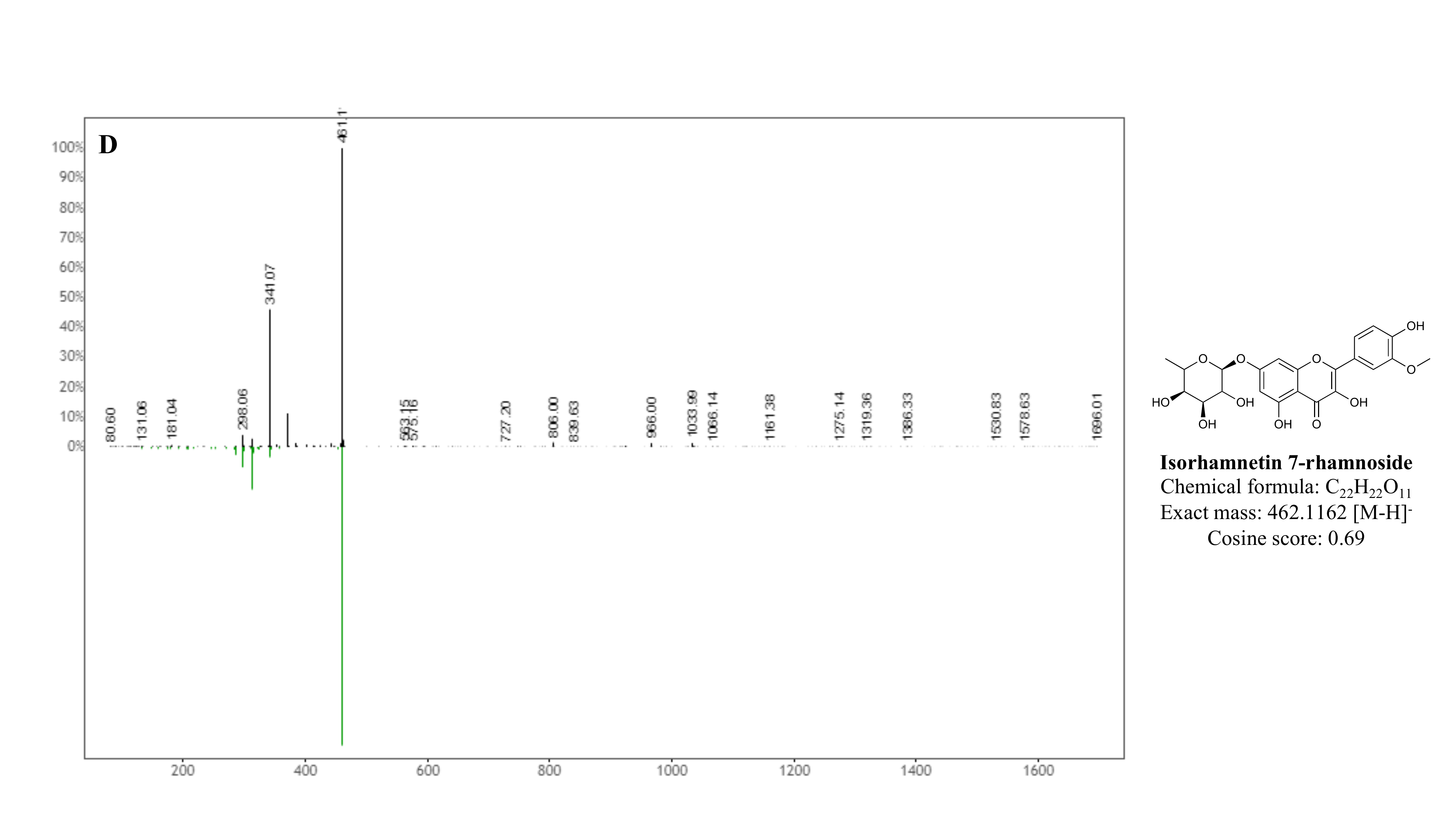

Supplement: Supplementary file 5 [file Image5.TIFF]

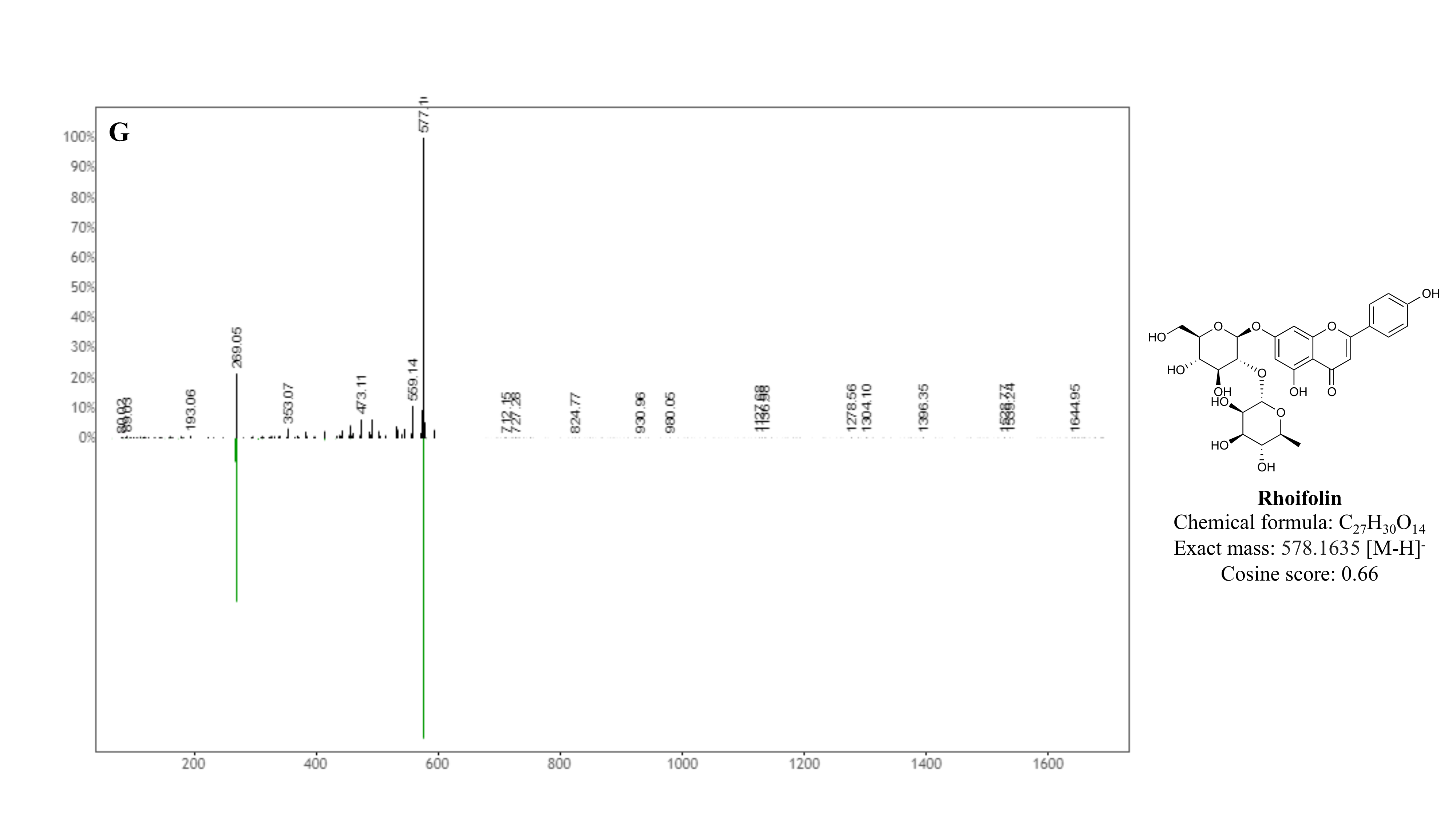

Supplement: Supplementary file 6 [file Image8.TIFF]

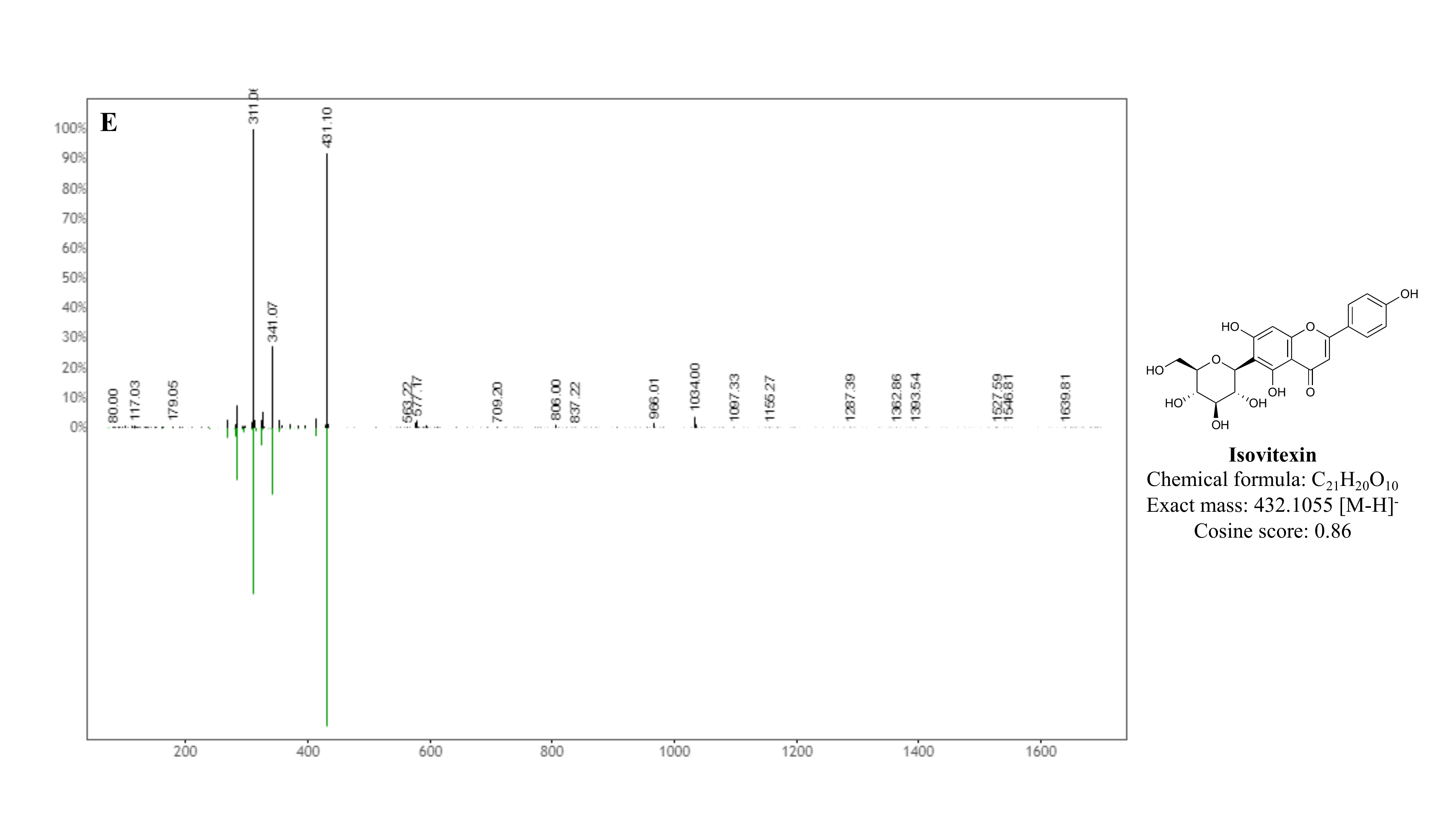

Supplement: Supplementary file 7 [file Image6.TIFF]

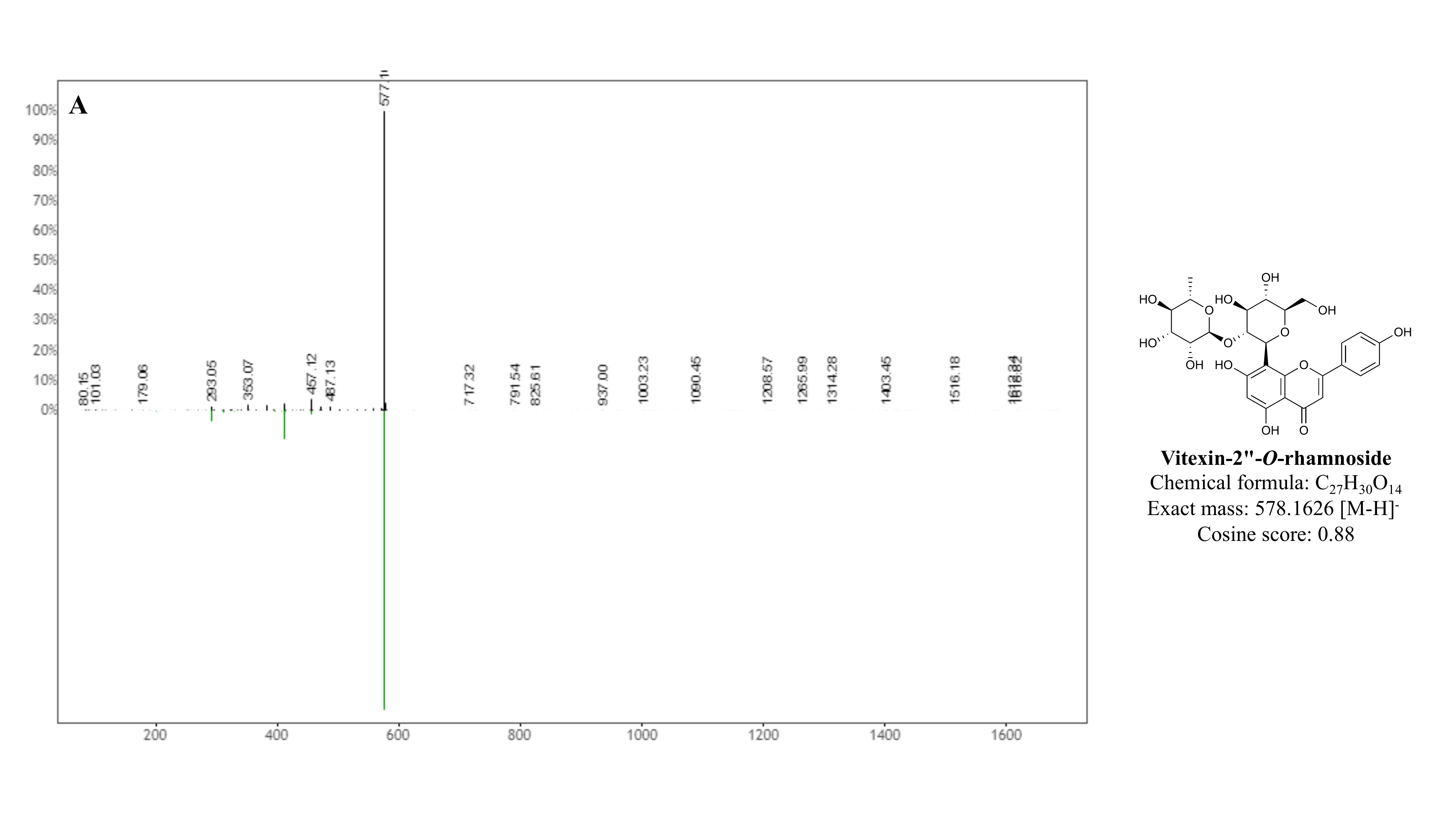

Supplement: Supplementary file 8 [file Image2.TIFF]

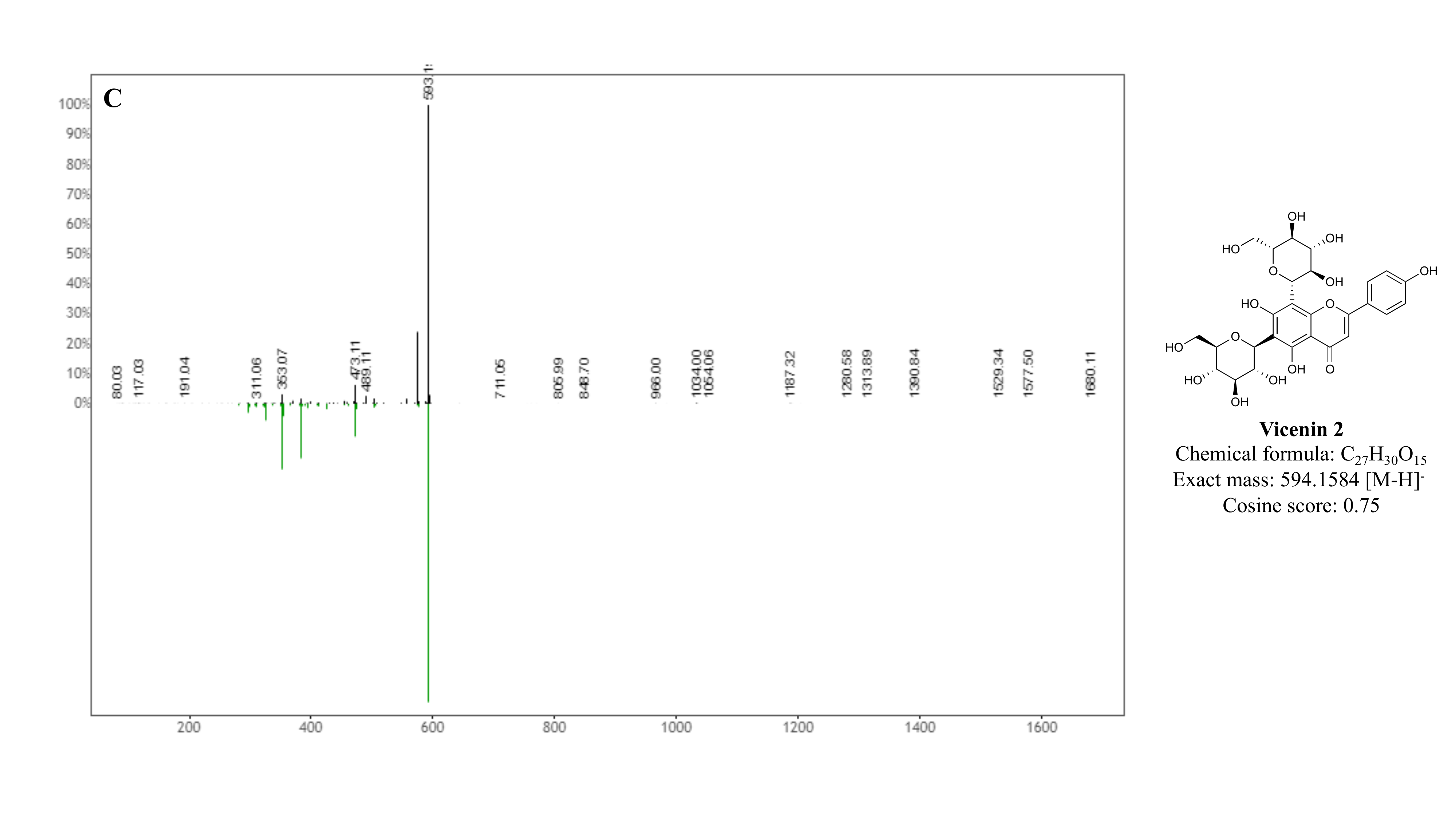

Supplement: Supplementary file 9 [file Image4.tiff]

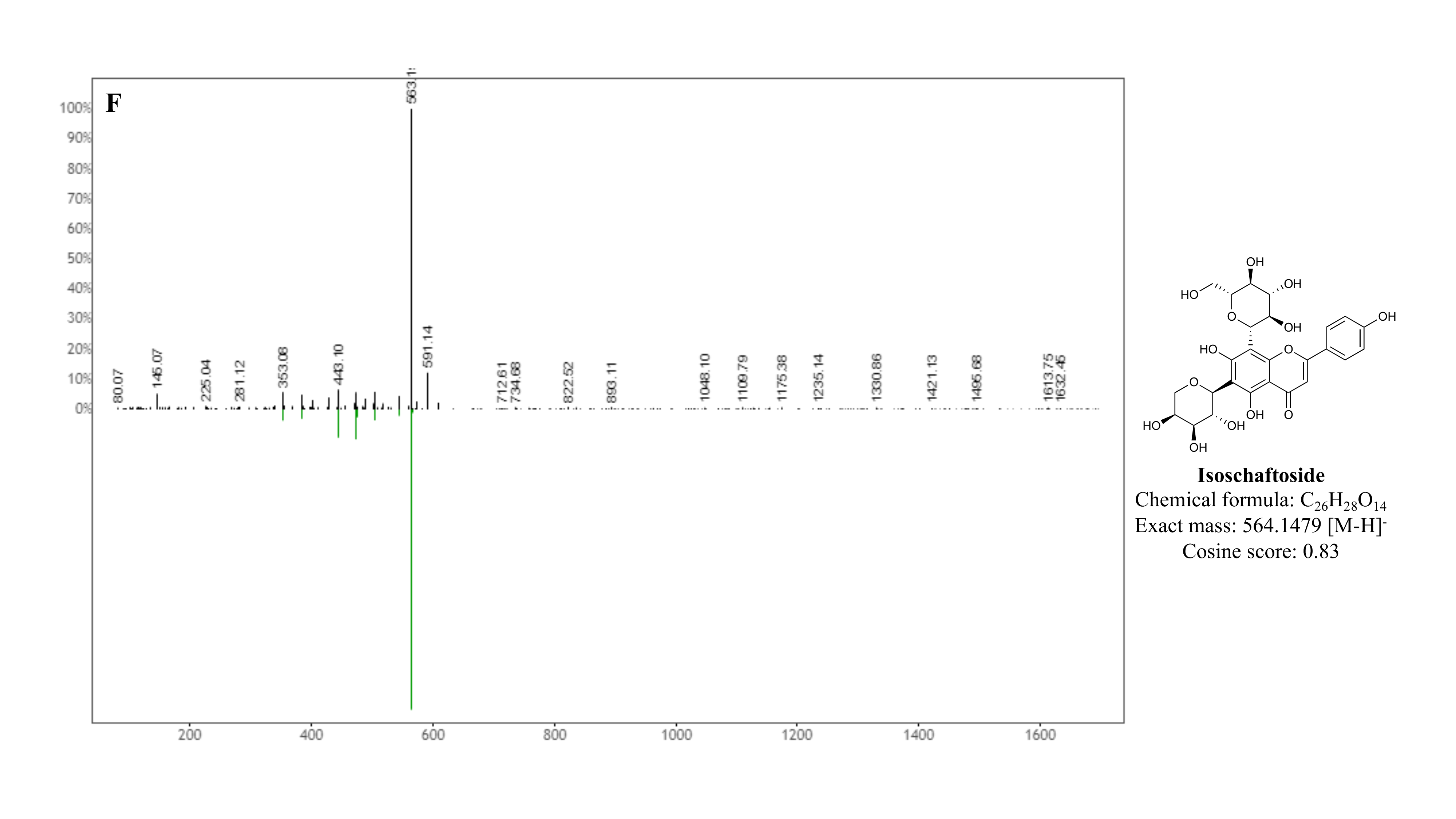

Supplement: Supplementary file 10 [file Image7.TIFF]
