# Supplementary material for: Untargeted metabolomics approach and molecular networking analysis reveal changes in chemical composition under the influence of altitudinal variation in bamboo species
Source: Front Mol Biosci. 2023 May 24;10:1192088. doi: 10.3389/fmolb.2023.1192088 (PMC10246775; doi:10.3389/fmolb.2023.1192088)
Supplement: Supplementary file 4 [file Table1.docx]

Supplementary Material

Untargeted metabolomics approach and molecular networking analysis reveal changes in chemical composition under the influence of altitudinal variation in bamboo species

Luis Carlos Chitiva, Hair Santiago Lozano-Puentes, Ximena Londoño, Tiago F. Leão, Mónica P. Cala, Eduardo Ruiz-Sanchez, Lucía Ana Díaz-Ariza, Juliet A. Prieto-Rodríguez, Ian Castro-Gamboa^*^, Geison M. Costa^*^

*** Correspondence:** Corresponding Author: [ian.castro@unesp.br](mailto:ian.castro@unesp.br); [modesticosta.g@javeriana.edu.co](mailto:modesticosta.g@javeriana.edu.co)

# Supplementary Figures and Tables

## Supplementary Figures

**Supplementary Figure 1.** PCA score plot including quality controls (QCs) and all samples from LC-QTOF-MS analysis.

**Supplementary Figure 2.** Dereplication of metabolites using Global Natural Social Molecular Networking (GNPS). The MS^2^ spectrum match feature of GNPS showing the similarity of fragment peaks of the experimental and library data. **(A)** Vitexin-2"-*O*-rhamnoside, **(B)** Saponarin, **(C)** Vicenin 2, **(D)** Isorhamnetin 7-rhamnoside, **(E)** Isovitexin, **(F)** Isoschaftoside, (**G)** Rhoifolin, and **(H)** Cyanidin 3-*O*-sophoroside.

## Supplementary Tables

**Supplementary Table 1.** List and information on the environmental variables of collection of the bamboo species used for the metabolomic study.

| **Plant scientific name** | **Sampling location** | **Collection date** | **AMAT (°C)** | **AMET (°C)** | **AMIT (°C)** | **MP (mm)** | **RH (%)** | **Longitude** | **Latitude** | **Altitude (m)** | **Voucher number** |
| --- | --- | --- | --- | --- | --- | --- | --- | --- | --- | --- | --- |
| *G. aculeata* E.Fourn. | Quindío, Montenegro | 12/2021 | 28.0 | 24.0 | 21.0 | 200-300 | 82.0 | -75.80102 | 4.52086 | 1256 | HPUJ-30736 |
| *G. amplexifolia* J.Presl | Quindío, Montenegro | 12/2021 | 28.0 | 24.0 | 21.0 | 200-300 | 82.0 | -75.80108 | 4.52083 | 1256 | HPUJ-30733 |
| *G. angustifolia* Kunth | Nariño, Tumaco | 01/2022 | 28.0 | 26.0 | 24.0 | 200-300 | 85.0 | -78.71333 | 1.56033 | 18 | HPUJ-30717 |
|  | Nariño, Tumaco | 01/2022 | 28.0 | 26.0 | 24.0 | 200-300 | 85.0 | -78.67738 | 1.54900 | 21 | HPUJ-30717 |
|  | Nariño, Ricaurte | 01/2022 | 24.0 | 20.0 | 16.0 | 400-600 | 75.0 | -78.05322 | 1.21986 | 1053 | HPUJ-30718 |
|  | Nariño, Ricaurte | 01/2022 | 24.0 | 20.0 | 16.0 | 400-600 | 75.0 | -78.03030 | 1.23202 | 1089 | HPUJ-30718 |
|  | Quindío, Montenegro | 12/2021 | 28.0 | 24.0 | 21.0 | 200-300 | 82.0 | -75.79944 | 4.51788 | 1256 | HPUJ-30731 |
|  | Cundinamarca, Pacho | 09/2020 | 31.5 | 25.5 | 23.5 | 100-200 | 82.5 | -74.19519 | 5.18127 | 1343 | HPUJ-30740 |
|  | Nariño, Samaniego | 01/2022 | 22.0 | 18.0 | 14.0 | 100-200 | 65.0 | -77.60819 | 1.31588 | 1478 | HPUJ-30719 |
|  | Nariño, Samaniego | 01/2022 | 22.0 | 18.0 | 14.0 | 100-200 | 65.0 | -77.59416 | 1.34527 | 1565 | HPUJ-30719 |
|  | Nariño, Samaniego | 01/2022 | 22.0 | 18.0 | 14.0 | 100-200 | 65.0 | -77.58619 | 1.33219 | 1588 | HPUJ-30719 |
|  | Nariño, San Lorenzo | 01/2022 | 20.0 | 15.0 | 12.0 | 200-300 | 71.0 | -77.16970 | 1.56840 | 1595 | HPUJ-30721 |
|  | Nariño, La Unión | 01/2022 | 21.0 | 18.0 | 12.0 | 200-300 | 63.0 | -77.12369 | 1.61061 | 1598 | HPUJ-30721 |
|  | Nariño, Consacá | 01/2022 | 22.7 | 19.5 | 14.5 | 100-200 | 66.0 | -77.48802 | 1.25369 | 1606 | HPUJ-30720 |
|  | Nariño, Samaniego | 01/2022 | 22.0 | 18.0 | 14.0 | 100-200 | 65.0 | -77.61022 | 1.30783 | 1606 | HPUJ-30719 |
|  | Nariño, La Unión | 01/2022 | 21.0 | 18.0 | 12.0 | 200-300 | 73.0 | -77.10830 | 1.61391 | 1610 | HPUJ-30721 |
|  | Nariño, La Unión | 01/2022 | 21.0 | 18.0 | 12.0 | 200-300 | 73.0 | -77.09472 | 1.61463 | 1631 | HPUJ-30721 |
|  | Nariño, San Lorenzo | 01/2022 | 20.0 | 15.0 | 12.0 | 200-300 | 71.0 | -77.20347 | 1.59688 | 1713 | HPUJ-30721 |
|  | Nariño, Sandoná | 01/2022 | 21.8 | 16.8 | 13.6 | 100-200 | 65.0 | -77.48088 | 1.32755 | 1720 | HPUJ-30719 |
|  | Nariño, Sandoná | 01/2022 | 21.8 | 16.8 | 13.6 | 100-200 | 65.0 | -77.48088 | 1.32755 | 1744 | HPUJ-30719 |
|  | Nariño, San Lorenzo | 01/2022 | 20.0 | 15.0 | 12.0 | 200-300 | 71.0 | -77.20711 | 1.58952 | 1779 | HPUJ-30721 |
|  | Nariño, San Lorenzo | 01/2022 | 20.0 | 15.0 | 12.0 | 200-300 | 71.0 | -77.20669 | 1.58786 | 1808 | HPUJ-30721 |
|  | Nariño, San Lorenzo | 01/2022 | 20.0 | 15.0 | 12.0 | 200-300 | 71.0 | -77.20700 | 1.58680 | 1826 | HPUJ-30721 |
|  | Nariño, Chachagüí | 01/2022 | 20.6 | 17.5 | 12.3 | 100-200 | 60.0 | -77.28186 | 1.37486 | 1857 | HPUJ-30722 |
|  | Nariño, San Lorenzo | 01/2022 | 20.0 | 15.0 | 12.0 | 200-300 | 71.0 | -77.20591 | 1.58616 | 1876 | HPUJ-30721 |
|  | Nariño, San Lorenzo | 01/2022 | 20.0 | 15.0 | 12.0 | 200-300 | 71.0 | -77.16610 | 1.56820 | 1930 | HPUJ-30721 |
|  | Nariño, San Lorenzo | 01/2022 | 20.0 | 15.0 | 12.0 | 200-300 | 71.0 | -77.16610 | 1.56820 | 1970 | HPUJ-30721 |
|  | Nariño, La Florida | 01/2022 | 20.0 | 16.0 | 10.0 | 100-200 | 64.0 | -77.41291 | 1.30511 | 2089 | HPUJ-30719 |
|  | Nariño, La Florida | 01/2022 | 20.0 | 16.0 | 10.0 | 100-200 | 64.0 | -77.41325 | 1.30041 | 2122 | HPUJ-30719 |
|  | Nariño, La Florida | 01/2022 | 20.0 | 16.0 | 10.0 | 100-200 | 64.0 | -77.40380 | 1.30005 | 2137 | HPUJ-30719 |
| *G. angustifolia* var. b*icolor* Londoño | Quindío, Montenegro | 04/2022 | 29.0 | 23.0 | 18.0 | 200-300 | 80.0 | -75.80056 | 4.52018 | 1256 | HPUJ-30735 |
| *G. angustifolia* Kunth biotype San Calixto | Quindío, Montenegro | 04/2022 | 29.0 | 23.0 | 18.0 | 200-300 | 80.0 | -75.80041 | 4.52063 | 1256 | HPUJ-30737 |
| *G. incana* Londoño | Putumayo, Mocoa | 12/2021 | 26.0 | 22.5 | 20.0 | 100-200 | 85.0 | -76.66477 | 1.17730 | 604 | HPUJ-30723 |
|  | Quindío, Montenegro | 12/2021 | 28.0 | 24.0 | 21.0 | 200-300 | 82.0 | -75.80013 | 4.52102 | 1256 | HPUJ-30723 |
| *G. superba* Huber | Quindío, Montenegro | 04/2022 | 29.0 | 23.0 | 18.0 | 200-300 | 80.0 | -75.80036 | 4.52100 | 1256 | HPUJ-30734 |
| *G. uncinata* Londoño & L.G.Clark | Quindío, Montenegro | 12/2021 | 28.0 | 24.0 | 21.0 | 200-300 | 82.0 | -75.80033 | 4.51816 | 1256 | HPUJ-30730 |
| *G. venezuelae* Munro | Quindío, Montenegro | 04/2022 | 29.0 | 23.0 | 18.0 | 200-300 | 80.0 | -75.80005 | 4.52057 | 1256 | HPUJ-30729 |
| *G. weberbaueri* Pilg. | Quindío, Montenegro | 04/2022 | 29.0 | 23.0 | 18.0 | 200-300 | 80.0 | -75.80057 | 4.52043 | 1256 | HPUJ-30738 |
| *B. vulgaris* Schrad. ex J.C. Wendl. | Cundinamarca, Pacho | 03/2021 | 31.5 | 25.5 | 23.5 | 100-200 | 82.5 | -74.19527 | 5.18138 | 1343 | HPUJ-30714 |
| *P. aurea* Rivière & C.Rivière | Quindío, Montenegro | 03/2021 | 28.0 | 24.0 | 21.0 | 200-300 | 82.0 | -74.19527 | 5.18138 | 1256 | HPUJ-30715 |

Annual Maximum Temperature - AMAT (°C), Annual Mean Temperature - AMET (°C), Annual Minimum Temperature - AMIT (°C), Monthly Precipitation - MP (Sampling Month) (mm), Relative Humidity - RH (%), Longitude, Latitude, and Altitude (m).
